# Age- and sex-dependent alterations of jejunal microbiota in Fischer 344 rats fed with a high-fructose, high-fat diet: depletion of *Lactobacillus intestinalis* in small bowel contents

**DOI:** 10.3389/fmicb.2026.1779112

**Published:** 2026-03-02

**Authors:** Sungchan Ha, Nayoung Kim, Chin-Hee Song

**Affiliations:** 1 Department of Health Science and Technology, Graduate School of Convergence Science and Technology, Seoul National University, Seoul, Republic of Korea; 2 Department of Internal Medicine, Seoul National University Bundang Hospital, Seongnam, Republic of Korea; 3 Research Center for Sex- and Gender-Specific Medicine, Seoul National University Bundang Hospital, Seongnam, Republic of Korea; 4 Department of Internal Medicine and Liver Research Institute, Seoul National University College of Medicine, Seoul, Republic of Korea

**Keywords:** age, high-fructose high-fat diet, microbiota, non-alcoholic fatty liver disease, sex, small bowel

## Abstract

**Introduction:**

Our previous research demonstrated that a high-fat diet (HFD) induced jejunal inflammation and hepatic steatosis, suggesting that small bowel microbiota contribute to these pathologies. This study investigated age- and sex-specific alterations in jejunal microbiota following a high-fructose, high-fat diet (HFHFD) in F344 rats.

**Methods:**

Six-week-old and two-year-old rats of both sexes were fed an HFHFD for 8 weeks, after which jejunal contents were collected for metagenomic analysis. Taxonomic profiling and linear discriminant analysis were performed, and Spearman’s rank correlation analysis was used to evaluate associations with jejunal inflammation and hepatic steatosis. Beta-diversity analysis was conducted to assess group separation. *In vitro*, HIEC-6 human intestinal epithelial cells were used to test the protective effect of *Lactobacillus intestinalis* under palmitic acid–induced lipotoxic stress.

**Results:**

HFHFD reduced the Firmicutes/Bacteroidetes ratio in young females and in aged rats of both sexes. Notably, *Lactobacillus intestinalis*—which supports barrier function—decreased in young males and aged females. In contrast, *Akkermansia muciniphila* increased across all HFHFD groups, particularly in young females and aged rats. *Bacteroides vulgatus* increased in aged HFHFD-fed rats of both sexes, while *Bacteroides caccae* was elevated in females across both age groups. Furthermore, the *Lactobacillus reuteri* group decreased only in young HFHFD rats. *L. intestinalis* and *L. reuteri* groups negatively correlated with jejunal inflammation and hepatic steatosis, whereas *B. caccae* and *A. muciniphila* showed positive correlations with both pathogenic phenotypes. Beta-diversity revealed a pronounced diet- and sex-dependent separation in young rats, which was attenuated in aged groups. In HIEC-6 cells, *L. intestinalis* significantly restored viability under palmitic acid–induced lipotoxic stress, though its conditioned medium did not.

**Discussion:**

Collectively, HFHFD induces age- and sex-dependent dysbiosis in the jejunum, and *L. intestinalis* may serve as a potential probiotic for metabolic dysfunction-associated steatotic liver disease.

## 1 Introduction

Metabolic dysfunction-associated steatosis (MASLD) affects approximately 30% of the global population, and its burden is expected to increase in parallel with rising obesity rates and shifts in dietary patterns (Wong et al., 2023; Younossi et al., 2023; Choi et al., 2024). The small bowel, the longest section of the gastrointestinal (GI) tract and the principal site of nutrient absorption, is an important contributor to liver pathology, including liver cirrhosis (Aoyama et al., 2013; Volk and Lacy, 2017). Although intestinal physiology influences metabolic liver injury (Cui et al., 2025), the specific contribution of the small bowel environment, particularly its role in nutrient absorption, mucosal permeability, and local microbiota, remains poorly understood in MASLD (Dumitru et al., 2025; Schnabl et al., 2025). Recently, our team showed an association between high-fructose high-fat diet (HFHFD) feeding, increased jejunal inflammation, and a reduction in the villus-to-crypt ratio, suggesting impaired intestinal function (Ha et al., 2025). In addition, jejunal inflammation was positively associated with hepatic steatosis, and increased claudin-2 expression showed a similar pattern, indicating a potential role of jejunal barrier dysfunction in MASLD pathogenesis (Ha et al., 2025). Growing evidences indicate that the gut microbiota plays a central role in human health and immune homeostasis, influencing not only GI disorders, such as inflammatory bowel disease (Odamaki et al., 2016) and colorectal cancer (Botteri et al., 2008), but also metabolic diseases, including diabetes (Cardinali et al., 2020) and obesity (Cuevas-Sierra et al., 2019). This gut microbiota is strongly influenced by dietary and lifestyle factors (Rinninella et al., 2023). Given these broad effects, characterization of gut microbial communities has become essential for understanding their functional impact on host physiology. Metataxonomic profiling using 16S rRNA sequencing has become a standard approach for characterizing microbial communities, and predictive tools, such as PICRUSt, enable functional inference based on Kyoto encyclopedia of genes and genomes (KEGG) orthology, providing insights into microbial metabolic pathways relevant to host physiology (Langille et al., 2013; Song et al., 2025).

The small bowel is a key site where dietary substrates interact with the resident microbiota (Delbaere et al., 2023); thus, alterations in jejunal microbial activity may directly affect the metabolic outputs. These absorbed nutrients and microbial metabolites from the small bowel are delivered to the liver via the portal vein (Fei et al., 2025). Cumulatively, we hypothesized that alterations in jejunal microbiota might influence hepatic metabolism and inflammation, contributing to MASLD progression. Therefore, we aimed to investigate sex- and age-specific differences in the jejunal microbiota of HFHFD-fed rats. Moreover, we attempted to identify specific species-level shifts in the jejunal microbial composition that might affect jejunal inflammation and MASLD.

## 2 Methods

### 2.1 Animals and diets

Six-week-old and 2-year-old male and female F344/NSIc rats were obtained from Orient Bio (Seoul, Korea) and maintained under specific pathogen-free conditions. Animals were housed at 23 °C with a 12-h light/dark cycle, and all animal procedures were conducted in accordance with the guidelines of the Institutional Animal Care and Use Committee of Seoul National University Bundang Hospital (approval no. BA-2305-367-005-01). Rats were assigned to one of two diets: a chow diet (3.85 kcal/g) as the control (CON), or a high-fat diet (5.24 kcal/g, 60% fat; Raonbio, D12492) and 20% fructose solution of water (Sigma-Aldrich, F0127) (HFHFD) (Lee et al., 2018; Choi et al., 2024). Eight experimental groups were established based on age, sex, and diet: 6-week-old males (CON *n* = 6; HFHFD *n* = 6), 6-week-old females (CON *n* = 6; HFHFD *n* = 6), 2-year-old males (CON *n* = 5; HFHFD *n* = 6), and 2-year-old females (CON *n* = 6; HFHFD *n* = 6). One aged male control rat died during the trial, resulting in a final sample size of *n* = 5 for the aged male CON group. This can occur in long-term studies using aged rodents. Over 8 weeks, caloric intake and body weight were recorded weekly. Specifically, body weight was measured once per week, and feed was replaced weekly. Food intake was calculated by subtracting the remaining food from the total amount provided at the beginning of the week. Similarly, water intake was recorded twice a week by measuring the remaining water, and a 20% fructose solution was replenished at each measurement. The caloric contribution of the fructose solution was calculated by multiplying the volume of solution consumed by the caloric content (4 kcal/g for fructose solution), as described in Ha et al. (2025). The caloric intake from the chow diet (3.85 kcal/g) and high-fat diet (5.24 kcal/g) was also calculated by multiplying the amount of food and water consumed by the corresponding caloric values. The total caloric intake from both the diets and the fructose solution was summed and divided by 7 to obtain a daily intake. At study completion, all rats were euthanized by CO₂ inhalation using a gradual-fill method in a dedicated chamber (approximately 5 L; CO₂ displacement rate ~30–40% chamber volume/min [1.5–2.0 L/min]). Death was confirmed by cessation of respiration, and jejunal tissues were promptly collected and frozen at −80 °C for downstream analyses.

### 2.2 Small bowel contents collection and metagenome sequencing

The collected jejunal tissues were placed in a petri dish and processed under cold conditions. Each sample was divided into two or three segments and gently rinsed with 1 mL of ice-cold Dulbecco’s Phosphate Buffered Saline (DPBS) (Biowest, L0615-500) using a syringe (Hecking et al., 2023). The wash solution was collected with a pipette and transferred to a sterile 2 mL tube. The tissue was then incised longitudinally, rinsed again with 1 mL of DPBS, and the wash was collected in a new 2 mL tube. This tube was centrifuged at 4 °C and 2000 rpm for 5 min, and the resulting supernatant was combined with the previously collected sample.

Genomic DNA was extracted from jejunal contents using the QIAamp DNA Mini Kit (Qiagen, 51,306) according to the manufacturer’s instructions. Subsequent amplification of the 16S rRNA V3–V4 region and metagenomic sequencing were performed as described previously (Choi et al., 2024; Song et al., 2025). Metagenome sequencing was conducted on the purified PCR product from the 16S rRNA amplicon at CJ Bioscience, Inc. (Seoul, South Korea). Primer sequences were trimmed using the CJ Bioscience in-house program, and nonspecific amplicons were identified using the HMMER program.

### 2.3 Microbiota analysis

Data processing and analysis were performed by CJ Bioscience following a previously described procedure (Song et al., 2025). Paired-end reads were assembled and subjected to quality control using an in-house bioinformatics pipeline that removed nonspecific, non-target, and chimeric amplicons. Sequencing quality metrics, including total raw reads, valid reads, and read length statistics, are provided in Supplementary Dataset S1 for further details. Taxonomic classification was performed against the EzBioCloud 16S rRNA database using USEARCH (version 8.1.1861_i86linux32), and operational taxonomic units (OTUs) were generated using UCLUST. For downstream analyses, reads were normalized to 28,783 reads per sample, and rarefaction curves were generated using EzBioCloud. Alpha- and beta-diversity analyses were performed to evaluate microbial community composition. Beta diversity was assessed using principal coordinate analysis (PCoA) based on the generalized UniFrac distance (*α* = 0.5) at the species level, a phylogeny-informed metric that balances the contributions of rare and abundant lineages. In generalized UniFrac, the parameter α controls the weight placed on abundant lineages (α = 1 corresponds to weighted UniFrac, while lower α values reduce dominance by highly abundant taxa); we used α = 0.5 as a commonly used robust setting (Chen et al., 2012). The relative abundance of the taxa was calculated to compare the abundance ratios among the groups. Graphical visualization of the selected taxa was performed using Prism software (GraphPad Software).

### 2.4 Taxonomic biomarker analysis and functional profiling of the small bowel contents microbiota

Linear discriminant analysis (LDA) effect size (LEfSe) was used to identify taxonomic biomarkers differentiating the microbiota composition among the diet groups at the species level. Group differences were assessed using the Kruskal–Wallis test (*p* < 0.05), and taxa that consistently differed among subgroups were confirmed using the pairwise Wilcoxon test. Features with a logarithmic LDA score > 2.0 were considered significant. Pathway analysis was performed using MinPath based on the KEGG orthology to predict the functional potential of the microbial community. Graphical visualization of the selected taxa was performed using Prism and RStudio software.

### 2.5 Cell and bacterial culture conditions

Human normal small intestinal epithelial cells (HIEC-6; ATCC, CRL-3266) were cultured in Opti-MEM (Gibco, #31985070) supplemented with 4% fetal bovine serum (FBS; Gibco, #16000044), GlutaMAX (Gibco, #35050061), 1 mM HEPES (Gibco, #15630080), and 10 ng/mL epidermal growth factor (EGF; Corning, #354052), and maintained at 37 °C in a humidified incubator with 5% CO₂. To model high-fat diet (HFD) conditions *in vitro*, palmitic acid (PA) (Sigma-Aldrich, P0500-10G) was used. PA was first dissolved in ethanol to prepare a 250 mM stock solution at 70 °C, then conjugated to 10% bovine serum albumin (BSA) to obtain an 8 mM PA–BSA solution by incubation at 37 °C overnight, followed by sterile filtration through a 0.22 μm filter (Knight et al., 2021). *L. intestinalis* (KCTC, 5052) was cultured in MRS broth (Difco, #288130) at 37 °C in a shaking incubator with 5% CO₂. For treatment, bacteria were prepared at 1 × 10^8^ CFU/mL in serum-free Opti-MEM (SFM). Conditioned medium (CM) was prepared by incubating bacteria in SFM at the same density (1 × 10^8^ CFU/mL), and the resulting CM was sterile filtered through a 0.22 μm filter.

HIEC-6 cells were seeded into 96-well plates at 1 × 10^4^ cells/well and allowed to attach for 24 h. Cells were then pretreated for 24 h with either live *L. intestinalis* (1 × 10^8^ CFU/mL) (Choi et al., 2024) or CM. To induce lipotoxic stress, PA was added in the presence of bacteria or CM at a final concentration of 400 μM for 12 h (Fatima et al., 2019; Ouyang et al., 2022). After PA exposure, the medium was removed, and cells were treated again with the corresponding bacteria or CM for an additional 24 h. Media were aspirated, cells were gently rinsed with DPBS (Biowest, L0615-500), and 100 μL/well of SFM was added. Cell viability was assessed using the CCK-8 assay (Dojindo, CK-04-11) according to the manufacturer’s instructions, and the absorbance was measured at 450 nm after 4 h of incubation. These experiments were repeated three times to check the reproducibility.

### 2.6 Statistical analysis

Statistical analyses were performed using SPSS software (version 18.0.0; IBM Corp., Armonk, NY, USA), except for the beta-diversity analysis. All analyses were conducted using nonparametric procedures that do not require equal group sizes, and the exact sample size used for each analysis is indicated in the corresponding Results and figure legends. Differences in microbial abundance ratio and alpha-diversity among groups were analyzed using the Kruskal–Wallis, followed by pairwise comparisons using the Mann–Whitney U-test. Correlations between microbial abundance ratios in jejunal contents and pathological phenotypes (jejunal inflammation score and hepatic steatosis [%]) obtained in our previous study (Ha et al., 2025) were determined using Spearman’s rank correlation (pairwise complete observations). Statistical significance was set at *p* < 0.05.

### 2.7 Accession number of data

All the datasets generated in this study can be found in the National Center for Biotechnology Information (NCBI) Sequence Read Archive (SRA) Database (PRJNA1393281).

## 3 Results

### 3.1 Diet-, age-, and sex-dependent alterations in the jejunal microbiota at the phylum level

**Figure 1:**
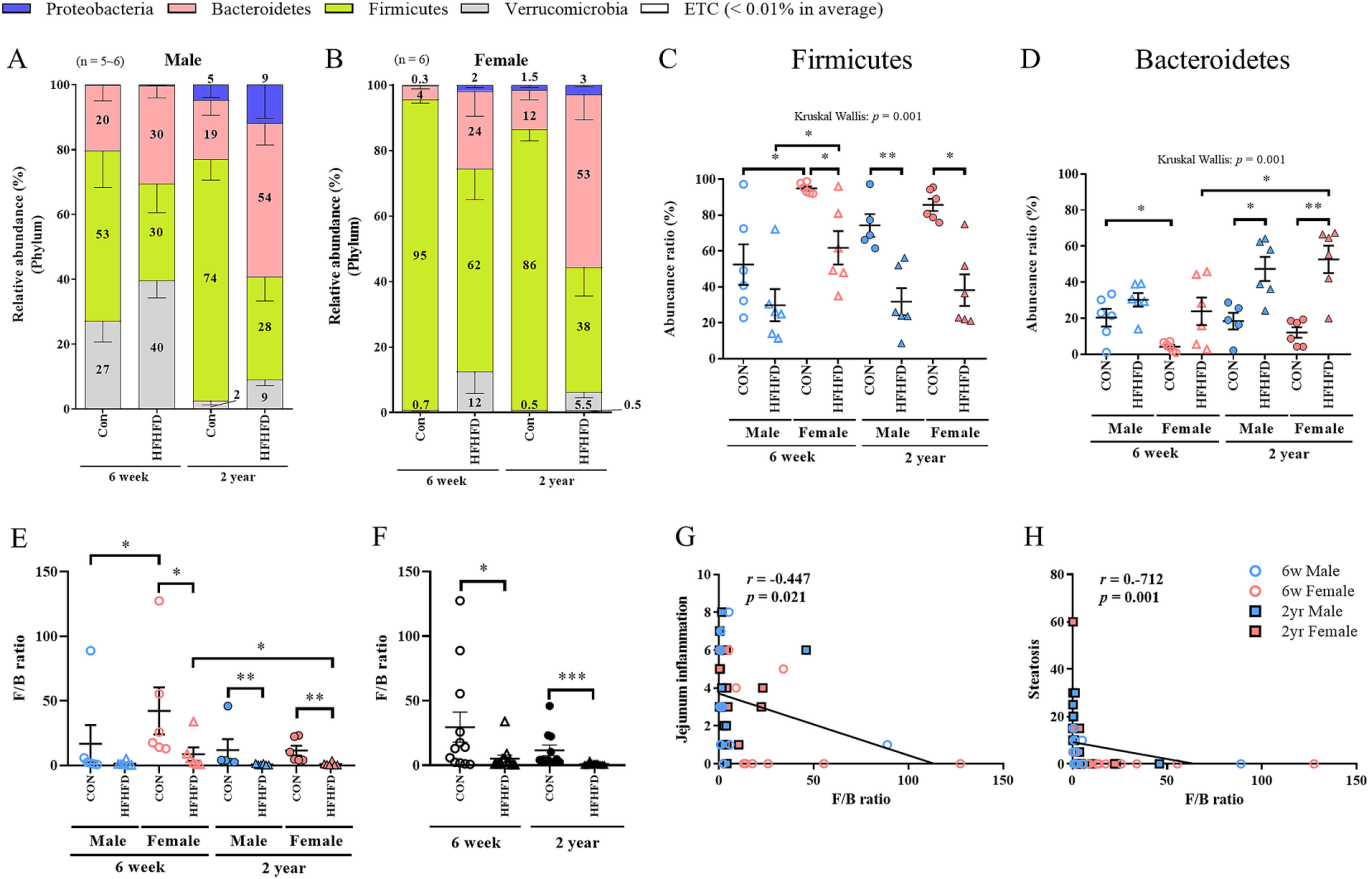
Taxonomic composition and Firmicutes/Bacteroidetes (F/B) ratio, and their correlations with hepatic steatosis and jejunal inflammation in the jejunal microbiota of rats fed different diets. Stacked bar plots show the relative taxonomic composition at the phylum level **(A,B)**. Scatter plots represent the relative abundance of **(C)** Firmicutes and **(D)** Bacteroidetes. **(E)** Comparison of F/B ratio according to age, sex, and diet. **(F)** Comparison of F/B regardless of sex. **(G)** Correlation between jejunal inflammation and F/B ratio. **(H)** Correlation between hepatic steatosis and F/B ratio. Scatter plot data are expressed as mean ± SEM. Statistical significance was determined using the Kruskal–Wallis test (*p*-values shown above the graphs) followed by the Mann–Whitney *U*-test (*p < 0.05, **p < 0.01, ***p < 0.001). Correlation was assessed using Spearman’s rank correlation (*n* = 6 per group, except for 2-year-old male CON, *n* = 5); 6w, 6 weeks; 2 yr., 2 years; CON, control; HFHFD, high-fructose high-fat diet.

At the phylum level, both male and female rats in the HFHFD group showed a decrease in Firmicutes and an increase in Bacteroidetes and Verrucomicrobia compared to the CON group (Figures 1A,B). When each group was analyzed separately, Firmicutes was significantly decreased in the young female HFHFD group, and its abundance ratio was reduced in both the male and female HFHFD groups (Figure 1C). In the young group, Firmicutes abundance was significantly higher in females than in males under both CON and HFHFD conditions (Figure 1C; Supplementary Figure 1B). Bacteroidetes abundance was significantly increased in both males and females in the aged HFHFD group (Figure 1D). In the young groups, Bacteroidetes abundance was significantly higher in male CON than in female CON rats (Figure 1D). Among females, the aged HFHFD group showed a markedly higher abundance than the young HFHFD group (Figure 1D). When the sexes were combined and the jejunal microbiota were compared according to diet and age, a consistent pattern was observed: Firmicutes decreased, whereas Bacteroidetes and Verrucomicrobia increased in the HFHFD groups (Supplementary Figure 1A). Proteobacteria showed an overall low relative abundance but tended to increase with advancing age within the same sex and dietary conditions (Figures 1A,B; Supplementary Figure 1E). Verrucomicrobia increased in response to HFHFD, showing a significant increase in the young female and aged male HFHFD groups (Supplementary Figure 1F). Moreover, young male CON rats exhibited a significantly higher Verrucomicrobia abundance than aged male CON rats and young female CON rats (Supplementary Figure 1F).

### 3.2 Alterations in the Firmicutes/Bacteroidetes ratio and its correlation with pathological phenotypes

The Firmicutes/Bacteroidetes (F/B) ratio decreased across all groups fed the HFHFD, with significant reductions observed in the young female and aged male and female HFHFD groups (Figure 1E). Although the young males in the HFHFD group showed a similar decrease, the difference did not reach statistical significance, likely because of the lower baseline ratio observed in the young males in the CON group. In the young CON group, females exhibited a significantly higher F/B ratio than males, indicating sex-dependent differences (Figure 1E). When sex was excluded as a variable and only diet and age were considered, the F/B ratio remained significantly lower in the HFHFD-fed rats than in the CON group (Figure 1F; Supplementary Figure 1C). Correlation analyses between the F/B ratio and previously measured pathological phenotypes (Ha et al., 2025) revealed a negative correlation, indicating that higher degrees of jejunal inflammation and hepatic steatosis, both elevated in the HFHFD group, were associated with a lower F/B ratio (Figures 1G,H).

### 3.3 Alterations in the relative abundance of major genus and species of the jejunal microbiota

**Figure 2:**
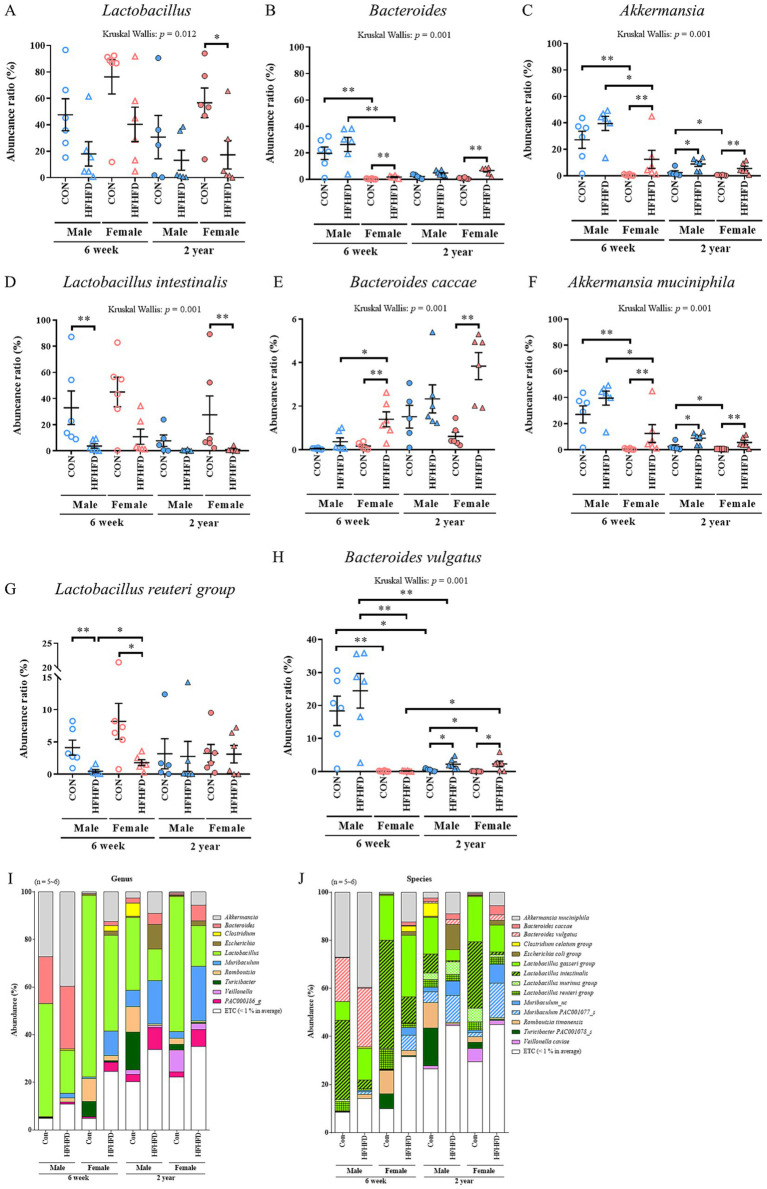
Alterations in the abundance ratio at the genus **(A–C)** and species **(D–H)** levels, along with major species, following exposure to a high-fat diet. Scatter plots show relative taxonomic abundance, with data expressed as mean ± SEM. **(A)** *Lactobacillus*; **(B)** *Bacteroides*; **(C)** *Akkermansia*; **(D)** *Lactobacillus intestinalis*; **(E)** *Bacteroides caccae*; **(F)** *Akkermansia muciniphila*; **(G)** *Lactobacillus reuteri group*; **(H)** *Bacteroides vulgatus*. In this dataset, *Akkermansia muciniphila* accounted for nearly all reads assigned to the genus *Akkermansia*, resulting in highly similar genus- and species-level abundance patterns. Stacked bar plots show the relative taxonomic composition at the genus and species level **(I,J)**. Statistical significance was determined using the Kruskal–Wallis test (*p*-values shown above the graphs) followed by the Mann–Whitney *U*-test (*p < 0.05, **p < 0.01, ***p < 0.001) (*n* = 6 per group, except for 2-year-old male CON, *n* = 5); 6w, 6 weeks; 2 yr., 2 years; CON, control; HFHFD, high-fructose high-fat diet.

At the genus level, changes in the abundance were mainly observed in *Lactobacillus*, *Bacteroides,* and *Akkermansia*. *Lactobacillus* showed a decreasing trend, with a significant reduction observed only in the aged female HFHFD group (Figure 2A). *Bacteroides* abundance increased significantly in female HFHFD rats compared to that in CON rats, regardless of age, and in the young groups, both male CON and HFHFD rats had significantly higher *Bacteroides* levels than their female counterparts (Figure 2B). In our data, the genus Akkermansia includes *Akkermansia muciniphila* and one unclassified species, with the abundance ratio of the unclassified species being very low. Therefore, the data for the genus Akkermansia and *Akkermansia muciniphila* are highlysimilar, leading to similar *p*-values and figures (Supplementary Dataset S2). *Akkermansia* tended to increase following HFHFD feeding and was significantly higher in young female HFHFD rats and in both sexes in the aged HFHFD group (Figure 2C). In both age groups, male CON rats showed significantly higher *Akkermansia* abundance than females, and the young male HFHFD group also exhibited higher *Akkermansia* abundance than the young female HFHFD group (Figure 2C).

Similar patterns were observed at the species level. *Lactobacillus intestinalis*, a commensal lactic acid bacterium residing in the small intestine (Wang et al., 2023), showed a decreasing tendency under HFHFD feeding, with significant reductions in young male HFHFD and old female HFHFD groups, indicating age-, sex-, and diet-dependent variations (Figure 2D). *Bacteroides caccae*, reported as an opportunistic pathogen (Schwenger et al., 2025), was significantly higher in female HFHFD rats than in the CON group, irrespective of age, whereas in the young HFHFD group, males exhibited significantly lower levels than females (Figure 2E). *Akkermansia muciniphila*, a beneficial commensal bacterium (Zhao et al., 2024), tended to increase in the HFHFD-fed rats, with significant elevations in young female HFHFD rats and in both sexes in the aged HFHFD groups (Figure 2F). Independent of age, male CON rats had a significantly higher *A. muciniphila* abundance than females, and young male HFHFD rats also showed higher levels than young female HFHFD rats (Figure 2F). The *Lactobacillus reuteri* group, known to be commensal (Lee et al., 2025), abundance significantly decreased in HFHFD-fed rats of both sexes at a young age, with male HFHFD rats showing lower levels than female rats, whereas no marked changes were observed in the aged groups (Figure 2G). *Bacteroides vulgatus*, an opportunistic pathogen (Bahitham et al., 2025), was notably more abundant in young male rats, with the young-male CON group displaying significantly higher levels than the female CON and aged male CON group. The effect of HFHFD was evident only in the aged groups, where *B. vulgatus* was significantly increased in both male and female HFHFD rats compared to their respective CON groups (Figure 2H). Finally, to provide a comprehensive overview of the jejunal microbial landscape, we analyzed the overall community composition at the genus and species levels (Figures 2I,J). A total of 483 genera and 1,624 species were identified, reflecting a highly diverse community. While the ‘ETC’ category (taxa with <1% average abundance) appeared relatively large due to this extensive diversity, the stacked bar plots clearly illustrate the dominant shifts in the community structure. These holistic profiles confirm that the individual taxonomic changes described above collectively contribute to a distinct remodeling of the rat jejunal microbiota, driven by the complex interplay of age, sex, and diet.

### 3.4 Correlations between jejunal microbial abundance and HFHFD-induced pathological phenotypes

**Figure 3:**
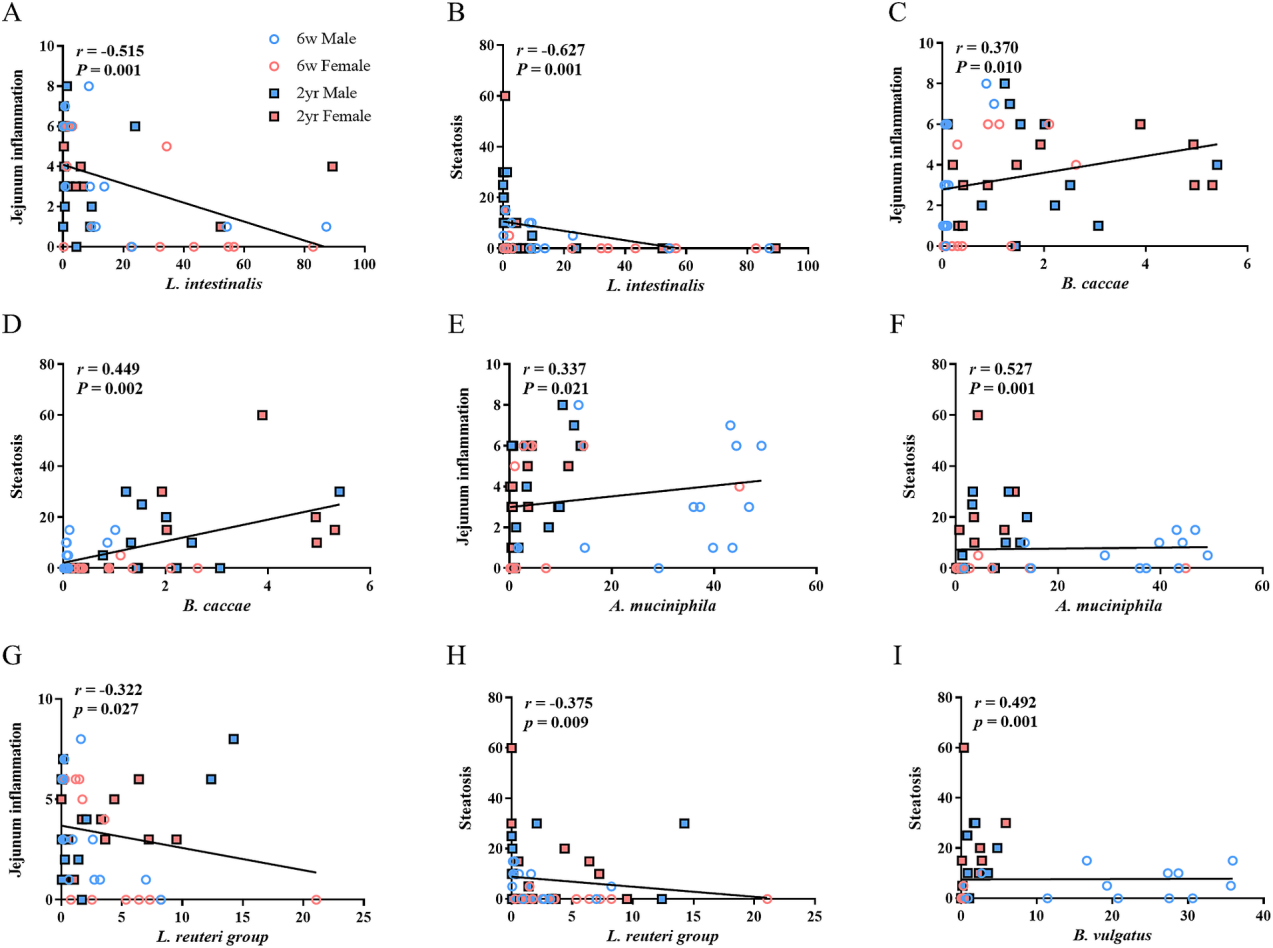
Correlations between histological parameters and bacterial abundance ratios in the jejunal microbiota of rats fed different diets. Scatter plots show correlations between histological features and the relative abundance of specific bacterial species. **(A)** Jejunal inflammation vs *Lactobacillus intestinalis*, **(B)** hepatic steatosis vs *L. intestinalis*, **(C)** jejunal inflammation vs *Bacteroides caccae*, **(D)** hepatic steatosis vs *B.caccae*, **(E)** jejunal inflammation vs *Akkermansia muciniphila*, **(F)** hepatic steatosis vs *A. muciniphila*, **(G)** jejunal inflammation vs *Lactobacillus reuteri* group, **(H)** hepatic steatosis vs *L. reuteri* group, **(I)** hepatic steatosis vs *Bacteroides vulgatus*. Statistical correlations were analyzed using Spearman’s rank correlation, and r- and p-values are indicated on the graphs (*n* = 6 per group, except for 2-year-old male CON, *n* = 5); 6w, 6 weeks; 2yr, 2 years.

Spearman’s rank correlation analysis was performed to determine whether the abundance ratio at the species level was associated with HFHFD-induced pathological phenotypes, including jejunal inflammation and hepatic steatosis (Ha et al., 2025). *L. intestinalis* was negatively correlated with jejunal inflammation, indicating that a lower abundance of *L. intestinalis* was associated with higher inflammatory scores (Figure 3A). Similarly, *L. intestinalis* negatively correlated with liver steatosis (Figure 3B). In contrast, *B. caccae* positively correlated with both jejunal inflammation and hepatic steatosis, with a higher *B. caccae* abundance corresponding to more severe pathological features (Figures 3C,D). *A. muciniphila* positively correlated with jejunal inflammation and hepatic steatosis, with increased abundance and progressive pathological phenotypes (Figures 3E,F). *L. reuteri* group showed patterns similar to those of *L. intestinalis*, with negative correlations indicating a reduced abundance under jejunal inflammation and steatosis (Figures 3G,H). *B. vulgatus* was positively correlated with hepatic steatosis (Figure 3I).

At the genus level, *Lactobacillus* negatively correlated with jejunal inflammation and liver steatosis (Supplementary Figures 2A,B). In contrast, *Bacteroides* positively correlated with these pathological phenotypes (Supplementary Figures 2C,D). The genus *Akkermansia* was positively correlated with jejunal inflammation and steatosis (Supplementary Figures 2E,F).

### 3.5 Age- and sex-dependent differences in beta diversity of the jejunal microbiota

**Figure 4:**
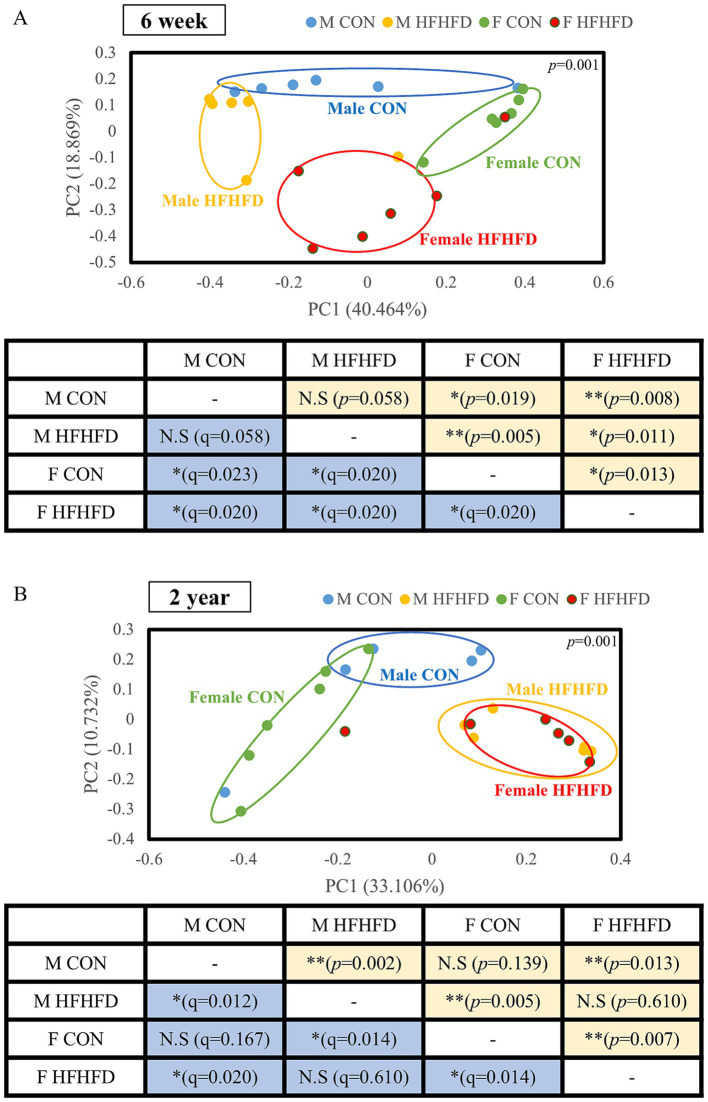
Distribution of jejunal microbiota according to diet, sex, and age in rats. Principal coordinate analysis (PCoA) plots illustrate the distribution of gut microbial communities according to dietary condition (CON *vs* HFHFD) and sex (male and female) in **(A)** 6-week-old and **(B)** 2-year-old rats. Each ellipse represents a 95% confidence interval for each group. Pairwise comparisons of microbial community composition between groups were performed using the PERMANOVA method on the EzBioCloud platform, and corresponding *p*- and *q*-values are summarized in the tables below each plot (all pairwise comparisons showed *p* = 0.001, as shown above the graphs). PC1 explains 40.464% of the variance, PC2 explains 18.869% of the variance for **(A)** 6-week-old rats and PC1 explains 33.106% of the variance, PC2 explains 10.732% of the variance for **(B)** 2-year-old rats (*n* = 6 per group, except for 2-year-old male CON, *n* = 5); CON, control; HFHFD, high-fructose high-fat diet.

Beta-diversity analysis using principal coordinate analysis (PCoA) revealed age- and sex-dependent separation patterns in the jejunal microbiota. In the young groups, all comparisons showed significant separation, except for the male CON and male HFHFD groups (*p* = 0.058, *q* = 0.058), which did not differ from each other (Figure 4A). Young female rats showed a clear separation between the CON and HFHFD groups (*p* = 0.013, *q* = 0.020), as well as from male CON rats (6-week-old F CON vs. M CON, *p* = 0.019, *q* = 0.023), indicating distinct effects of both diet and sex on the microbial community structure (Figure 4A). In contrast, the sex-dependent separation observed in young rats was diminished in aged rats (Figure 4B). Although both male and female rats showed a significant separation between the CON and HFHFD conditions (2-year-old M CON vs. M HFHFD, *p* = 0.002, *q* = 0.012; 2-year-old F CON vs. FHFHFD, *p* = 0.005, *q* = 0.014), the difference between male CON and female CON was no longer significant (*p* = 0.139, *q* = 0.167), and no separation was observed between the male and female HFHFD groups (*p* = 0.610, *q* = 0.610, Figure 4B). These findings indicate that sex-dependent differences in the microbiota structure present at a young age are attenuated with age.

When sex was analyzed independently, male rats showed no significant differences between the young CON and young HFHFD groups (*p* = 0.067, *q* = 0.067), whereas all other comparisons showed significant differences (Supplementary Figure 3A). In female rats, both diet and age contributed to a significant separation across all group comparisons, suggesting a stronger response of the female microbiota to HFHFD and aging (Supplementary Figure 3B). Alpha-diversity indices, including OTUs, Chao1, Shannon index, and phylogenetic diversity, did not show significant differences among the groups, as the values across the groups were highly variable (Supplementary Figures 2C–F).

### 3.6 LEfSe-based taxonomic biomarkers revealed diet-, age-, and sex-dependent alterations in the jejunal microbiota

**Figure 5:**
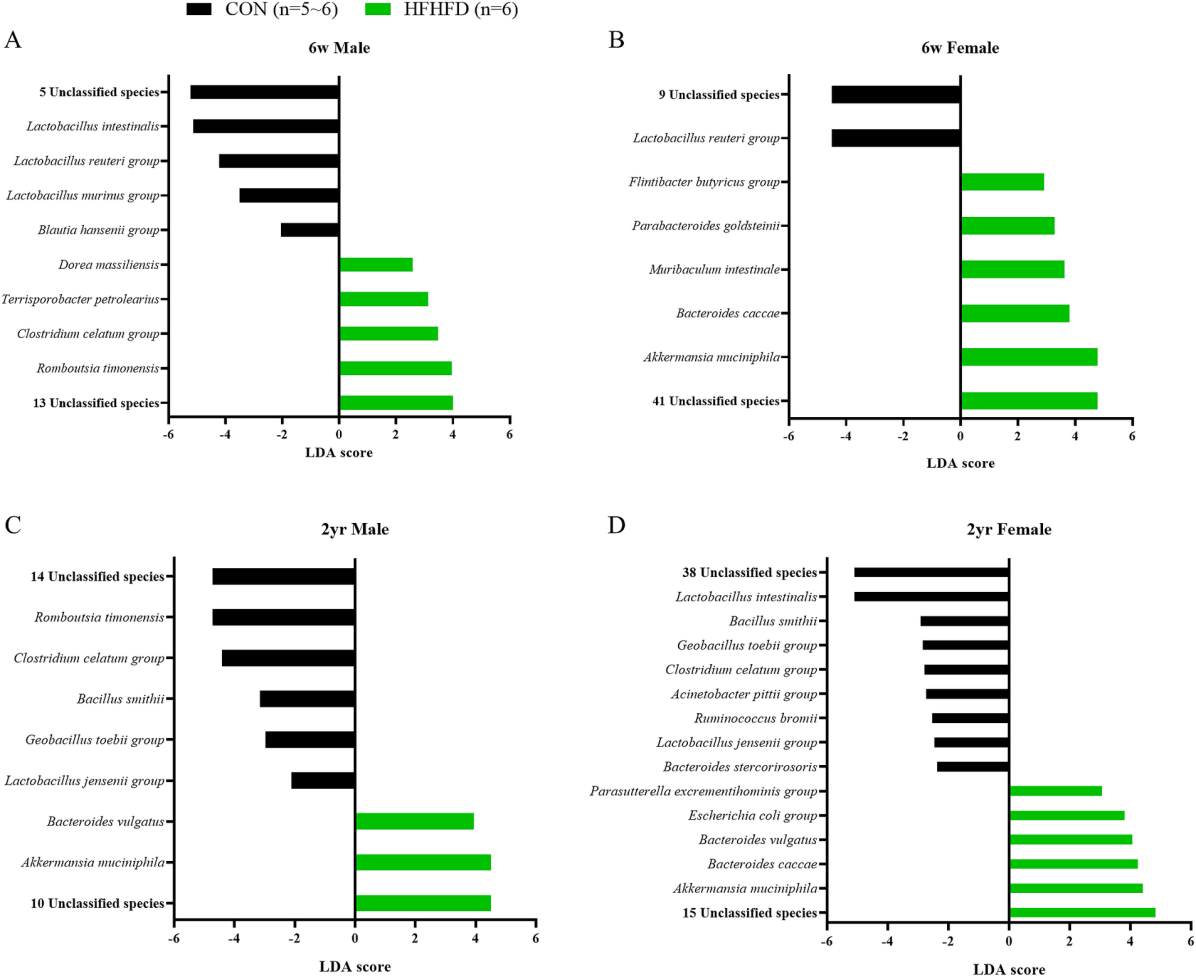
Differentially abundant bacterial species identified by LEfSe analysis in the jejunal microbiota of rats fed a control diet or HFHFD. LEfSe (Linear Discriminant Analysis Effect Size) was performed at the species level to identify significantly enriched bacterial taxa between the two dietary groups. Bar plots indicate bacterial species with LDA scores exceeding the threshold (LDA score > 2.0), representing those differentially abundant in each group. **(A)** 6-week-old males, **(B)** 6-week-old females, **(C)** 2-year-old males, **(D)** 2-year-old females. The length of each bar represents the effect size (LDA score) (*n* = 6 per group, except for 2-year-old male CON, *n* = 5); 6w, 6 weeks; 2 yr., 2 years; CON, control; HFHFD, high-fructose high-fat diet.

Using LEfSe analysis, we identified taxonomic biomarkers that were differentially enriched according to diet with clear age- and sex-dependent patterns. *A. muciniphila* was enriched in all HFHFD groups except young male rats, showing a consistently higher abundance in young female HFHFD rats and in both sexes of the aged HFHFD groups. In the young CON groups, both males and females showed significantly higher levels of *L. reuteri* than their HFHFD counterparts, indicating that this species is a shared biomarker of the CON condition (Figures 5A,B; Table 1). In the aged CON group, *Bacillus smithii*, *Lactobacillus jensenii* group, and *Geobacillus toebii* were enriched, representing age-associated biomarkers present only under non-HFHFD conditions (Figures 5C,D; Table 1). Conversely, among the HFHFD-fed rats, *B. vulgatus* was consistently enriched in both males and females in the aged groups. In addition, an unclassified species, *PAC001065_s*, was uniformly enriched across all HFHFD groups, indicating a diet-driven increase in specific, potentially opportunistic taxa (Table 1). Several additional biomarkers appeared in an age- or sex-specific manner. However, these species were restricted to individual comparisons and did not form consistent patterns across groups.

**Table 1:** Overlapping diet-dependent taxonomic biomarkers (CON vs. HFHFD) identified by LEfSe (LDA > 2.0).

Group (Diet/Age)	Control	High-fructose high-fat diet	
6-week (both sexes)	*Lactobacillus reuteri group* AB626927_s	3 unclassified species	PAC001065_s
2-year (both sexes)	*Bacillus smithii* *Lactobacillus jensenii group* *Geobacillus toebii group* FJ880879_s	*Bacteroides vulgatus* 2 unclassified species	
Male (both ages)	-	-	-
Female (both ages)	-	3 unclassified species	-

**Figure 6:**
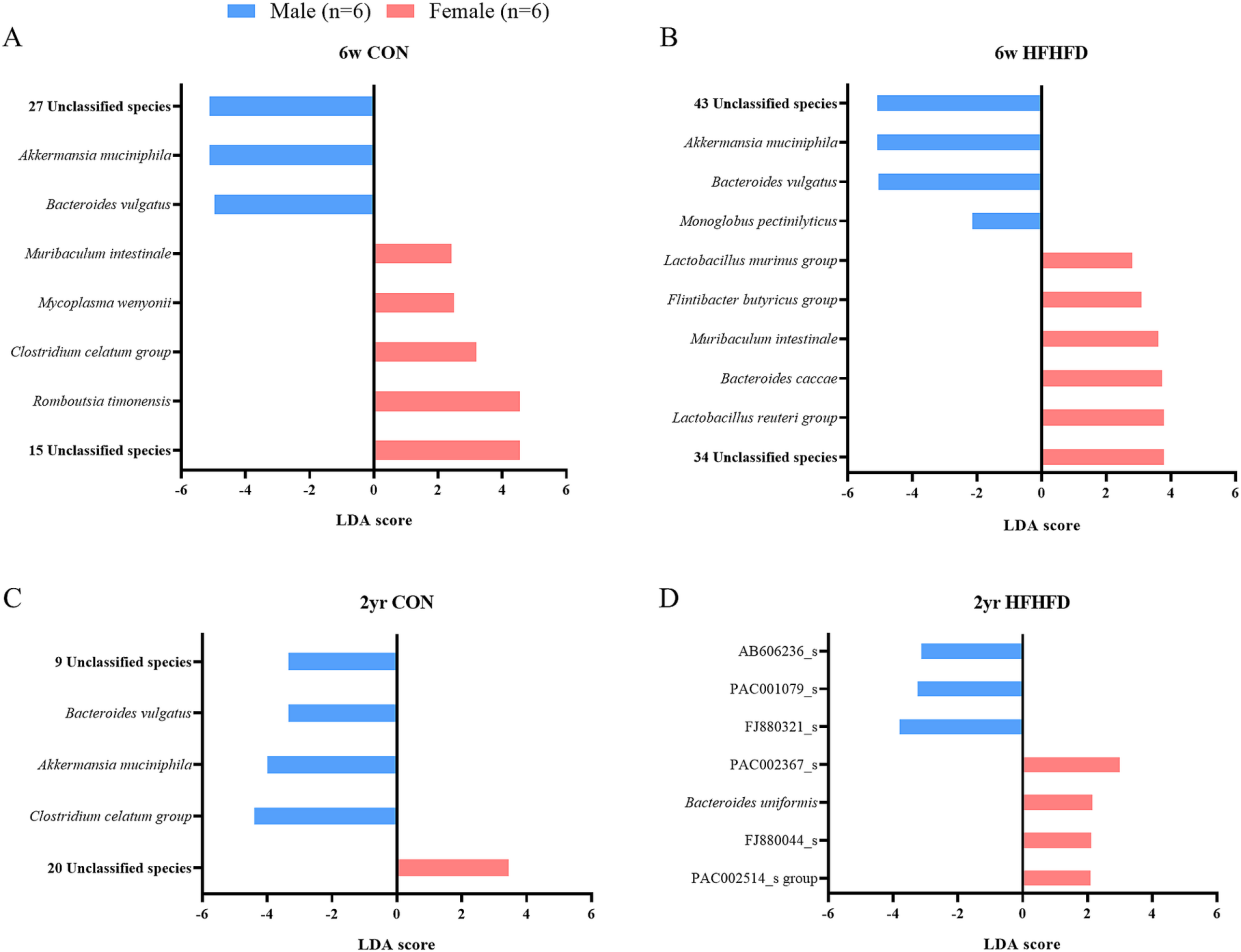
Differentially abundant bacterial species identified by LEfSe analysis in the jejunal microbiota of male and female rats within each diet and age group. LEfSe (Linear Discriminant Analysis Effect Size) was performed at the species level to identify sex-dependent differences in bacterial abundance under each dietary condition. Bar plots show bacterial species with LDA scores exceeding the threshold (LDA score > 2.0), representing those differentially abundant between males and females. **(A)** 6-week-old CON, **(B)** 6-week-old HFHFD, **(C)** 2-year-old CON, **(D)** 2-year-old HFHFD. The length of each bar represents the effect size (LDA score) (*n* = 6 per group, except for 2-year-old male CON, *n* = 5); 6w, 6 weeks; 2 yr., 2 years; CON, control; HFHFD, high-fructose high-fat diet.

To investigate the sex-dependent differences, we performed pairwise comparisons within each age and diet group. In the young male CON, young male HFHFD, and aged male CON groups, *A. muciniphila*, *B. vulgatus*, and the two unclassified species were significantly enriched compared to their female counterparts (Figures 6A–C). In addition, both young male CON and HFHFD rats showed a higher abundance of 21 overlapping unclassified species than females (Table 2). Conversely, young female rats exhibited higher levels of *Muribaculum intestinale* and the four unclassified species than male rats (Figures 6A,B; Table 2). Notably, the *Clostridium celatum* group displayed a sex- and age-dependent pattern, being enriched in young female CON rats and aged male CON rats (Figures 6A,C).

**Table 2:** Overlapping sex-dependent taxonomic biomarkers identified by LEfSe across diet and age (LDA > 2.0).

Group (Sex/Age)	Male	Female
6-week (both diets)	21 unclassified species	*Muribaculum intestinale* 4 unclassified species
2-year (both diets)	-	2 unclassified species

**Figure 7:**
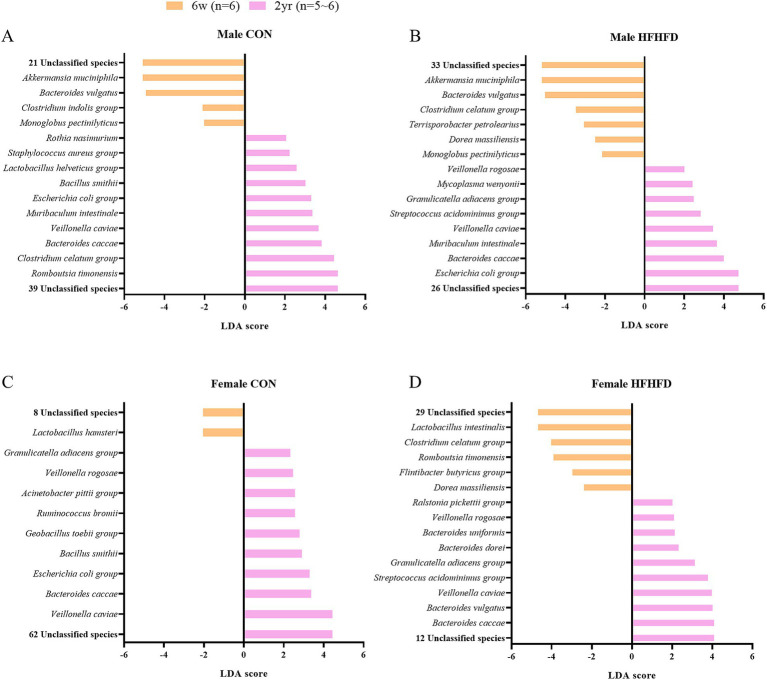
Differentially abundant bacterial species identified by LEfSe analysis in the jejunal microbiota of rats of different ages within each diet and sex group. LEfSe (Linear Discriminant Analysis Effect Size) was performed at the species level to identify age-related differences in bacterial abundance between 6-week-old and 2-year-old rats under each dietary condition. Bar plots show bacterial species with LDA scores exceeding the threshold (LDA score > 2.0), representing those differentially abundant between age groups. **(A)** Male CON, **(B)** male HFHFD, **(C)** female CON, **(D)** female HFHFD. The length of each bar represents the effect size (LDA score) (*n* = 6 per group, except for 2-year-old male CON, *n* = 5); 6w, 6 weeks; 2 yr., 2 years; CON, control; HFHFD, high-fructose high-fat diet.

To assess age-related differences, we compared young and aged rats irrespective of sex. In the CON group, *AB626927_s* was specifically enriched in young rats, whereas *Bacillus smithii* and eight unclassified species were enriched in aged CON rats (Table 3). Among the HFHFD-fed rats, *Streptococcus acidominimus* group, a known pathogen (Wu et al., 2014), was consistently enriched in aged rats, regardless of sex (Figures 7B,D; Table 3). Across all age groups, *B. caccae*, *Veillonella caviae*, and four unclassified species were elevated (Figures 7A–D; Table 3). Furthermore, young male rats showed a higher abundance of *A. muciniphila*, *B. vulgatus*, *Monoglobus pectinilyticus*, and 11 unclassified species independent of diet (Figures 7A,B; Table 3), whereas aged male rats showed higher levels of *M. intestinale* and nine unclassified species (Figures 7A,B; Table 3).

**Table 3:** Overlapping age-dependent taxonomic biomarkers identified by LEfSe across sex and diet (LDA > 2.0).

Group (Age/Diet)	6-week (both sexes)	2-year (both sexes)	
CON (both sexes)	AB626927_s	*Bacillus smithii* 8 unclassified species	*Bacteroides caccae* *Veillonella caviae* 4 unclassified species
HFHFD (both sexes)	*-*	*Streptococcus acidominimus group*	
Male (both diets)	*Akkermansia muciniphila* *Bacteroides vulgatus* *Monoglobus pectinilyticus* 11 unclassified species	*Muribaculum intestinale* 9 unclassified species	
Female (both diets)	-	2 unclassified species	

To further characterize taxonomic biomarkers that were not shared across comparisons, non-overlapping taxa were classified according to their reported ecological roles as commensals, opportunistic pathogens, or functionally uncharacterized pathogens. Commensal species are described as beneficial members of the gut microbiota, contributing to maintaining intestinal homeostasis. Opportunistic pathogens are now identified as species that may cause disease under certain conditions, such as dysbiosis or when the host’s immune system is compromised. In comparison by sex, in the young CON group, male rats showed a higher abundance of commensal *A. muciniphila* and one opportunistic pathogen, *B. vulgatus*, whereas female rats exhibited enrichment of three commensal species (*Muribaculum intestinale, Clostridium celatum, Romboutsia timonensis*) together with one opportunistic pathogen, *Mycoplasma wenyonii* (Figure 6A). In the young HFHFD group, males were characterized by the enrichment of two commensal species *(A. muciniphila, Monoglobus pectinilyticus)* and one opportunistic pathogen (*B. vulgatus*), whereas females showed a higher abundance of four commensal species (*Lactobacillus murinus, Flintibacter butyricus, M. intestinale, L. reuteri*) and one opportunistic pathogen (*B. caccae*) (Figure 6B). In the aged CON group, males exhibited an enrichment of two commensal species (*A. muciniphila, C. celatum*) along with one opportunistic pathogen (*B. vulgatus*). In contrast, in the aged HFHFD groups, females showed a higher abundance of the commensal species *Bacteroides uniformis* (Figures 6C,D). Taken together, the sex-dependent taxonomic biomarkers indicated reduced commensal abundance in young males, whereas in aged rats, commensal enrichment was relatively reduced in females.

### 3.7 Age-, sex-, and diet-dependent modulation of KEGG-based predicted metabolic and non-metabolic functional pathways

**Figure 8:**
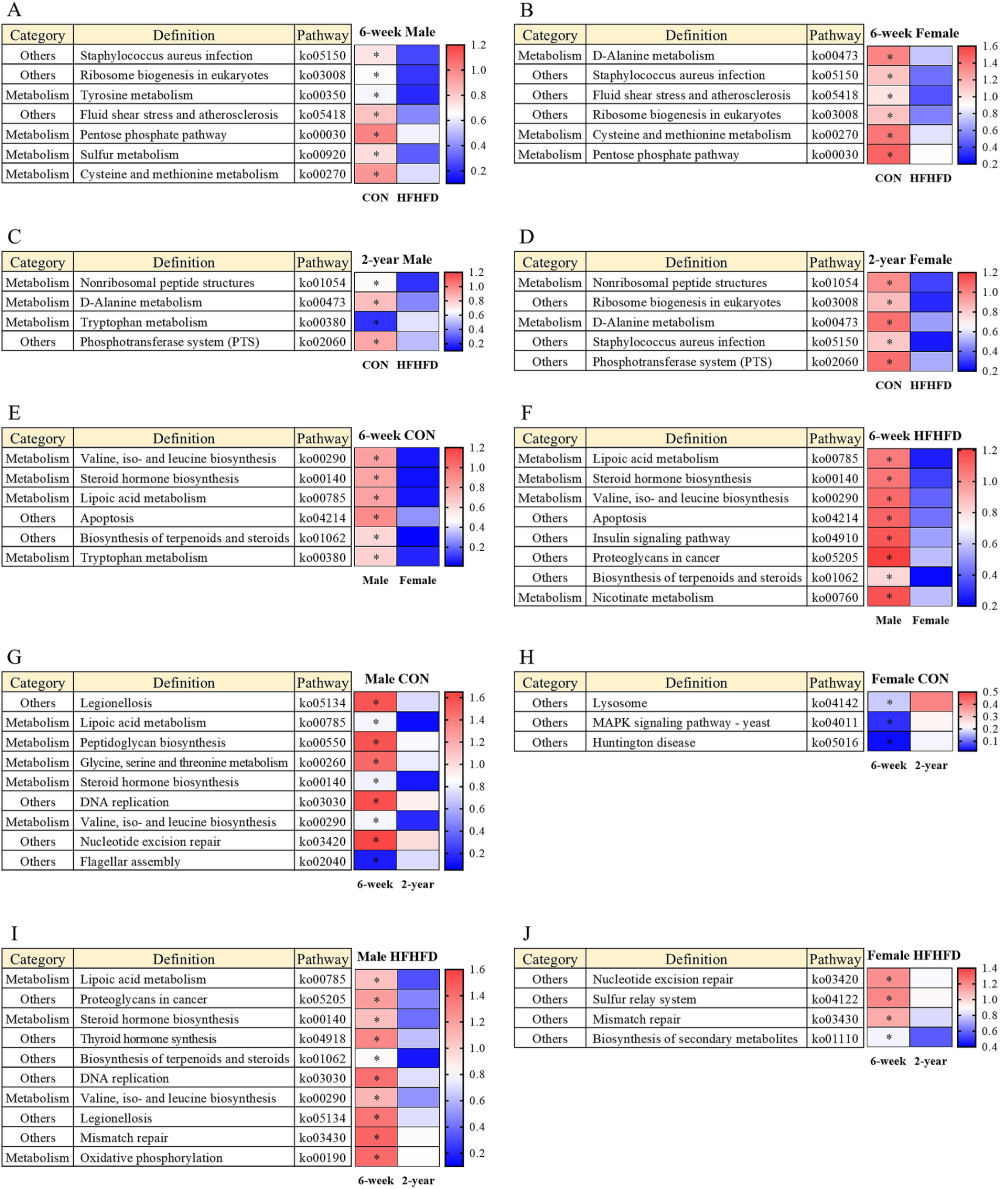
Functional biomarkers predicted by LEfSe analysis based on KEGG pathway classification in the jejunal microbiota of rats. LEfSe (Linear Discriminant Analysis Effect Size) was used to identify significantly different functional biomarkers inferred from KEGG pathway analysis. Heatmaps display pathways with significantly different LDA scores between the indicated groups (*p < 0.05, **p < 0.01). **(A–D)** Comparison between CON and HFHFD groups in **(A)** 6-week-old males, **(B)** 6-week-old females, **(C)** 2-year-old males, and **(D)** 2-year-old females. **(E,F)** Comparison between males and females within each dietary condition: **(E)** 6-week CON and **(F)** 6-week HFHFD. **(G–J)** Comparison between 6-week-old and 2-year-old rats within each sex and dietary group: **(G)** male CON, **(H)** male HFHFD, **(I)** female CON, and **(J)** female HFHFD. Color intensity represents the relative LDA score of each KEGG pathway category (*n* = 6 per group, except for 2-year-old male CON, *n* = 5); 6w, 6 weeks; 2 yr., 2 years; CON, control; HFHFD, high-fructose high-fat diet.

Predictive functional profiling was performed based on KEGG pathways, and representative functional differences were visualized using heatmaps and scatter plots. All comparisons were conducted in a pairwise manner, where deeper red indicated higher functional abundance and deeper blue indicated lower abundance. In young males, the predicted pathway abundances of tyrosine metabolism (ko00350), the pentose phosphate pathway (ko00030), sulfur metabolism (ko00920), and cysteine and methionine metabolism (ko00270) were significantly lower in the HFHFD group than in the CON group (Figure 8A). Young females exhibited similar reductions in ko00270 and ko00030, and d-alanine metabolism (ko00473) also showed decreased predicted pathway abundance (Figure 8B). In the non-metabolism (“others”) category, *Staphylococcus aureus* infection (ko05150), ribosome biogenesis in eukaryotes (ko03008), and fluid shear stress and atherosclerosis (ko05418) were significantly higher in CON in both young males and females (Figures 8A,B). In the aged group, both males and females showed a higher predicted pathway abundance of D-alanine metabolism (ko00473) and non-ribosomal peptide structures (ko01054) in the CON group than in the HFHFD group (Figures 8C,D). In the other category, the phosphotransferase system (ko02060) was commonly reduced under HFHFD in both sexes. Aged males demonstrated a significant reduction in tryptophan metabolism (ko00380) under CON conditions (Figure 8C).

To evaluate sex-dependent differences, comparisons within the young groups revealed that males exhibited higher predicted pathway abundances for valine, isoleucine, and leucine biosynthesis (ko00290), steroid hormone biosynthesis (ko00140), and lipoic acid metabolism (ko00785) than females in both the CON and HFHFD groups (Figures 8E,F). Tryptophan metabolism (ko00380) was lower in young CON females, whereas nicotinate metabolism (ko00760) was significantly lower in young HFHFD females (Figures 8E,F). In the other category, apoptosis (ko04214) and biosynthesis of terpenoids and steroids (ko01062) were consistently lower in females. In young HFHFD rats, the predicted pathway abundance of the insulin signaling pathway (ko04910) and proteoglycans in cancer (ko05205) was also significantly lower in females. Interestingly, no sex-dependent differences were observed in either the CON or HFHFD groups at 2 years of age, indicating that sex-specific functional distinctions were diminished in aged rats.

Next, age-dependent differences were assessed. In the metabolism category, peptidoglycan biosynthesis (ko00550), ko00785, ko00260, ko00140, and ko00290 were significantly lower in aged male CON rats than in young male CON rats (Figure 8G). In the other category, legionellosis (ko05134), DNA replication (ko03030), and nucleotide excision repair (ko03420) exhibited higher predicted pathway abundance in young males, whereas flagellar assembly (ko02040) was lower in young males (Figure 8G). In female CON rats, no age-related differences were observed in metabolism-related pathways. However, in the other categories, lysosomes (ko04142), Mitogen-Activated Protein Kinase signaling pathway (ko04011), and Huntington’s disease (ko05016) were significantly higher in the aged group (Figure 8H). In HFHFD male rats, age-dependent changes in metabolism pathways were similar to those observed in male CON rats: ko00785, ko00140, and ko00290 were significantly lower in aged rats (Figure 8I). Additionally, Oxidative phosphorylation (ko00190) was significantly higher in the young group (Figure 8I). In the other category, ko05134 and ko03030 remained higher in young male rats, consistent with CON rats, and thyroid hormone synthesis (ko04918), mismatch repair (ko03430), ko05205, and ko01062 levels were significantly elevated in young HFHFD male rats (Figure 8I). Metabolic pathways showed no age-dependent differences in female HFHFD rats. However, nucleotide excision repair (ko03420), sulfur relay system (ko04122), biosynthesis of secondary metabolites (ko01110), and ko03430 were significantly more abundant in young females than in aged females (Figure 8J).

### 3.8 Effects of *Lactobacillus intestinalis* and its CM on PA-induced high-fat diet condition in HIEC-6 cells

**Figure 9:**
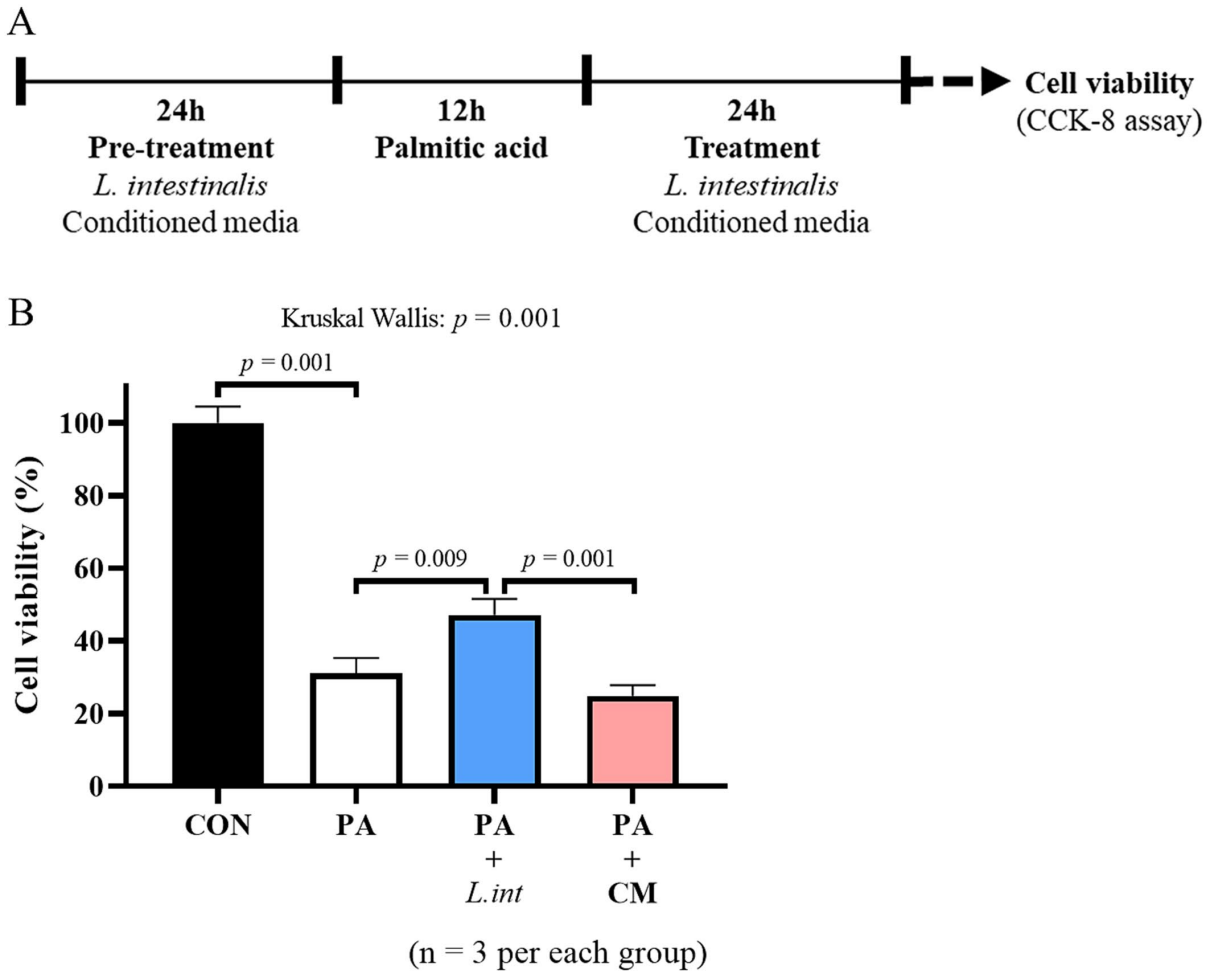
*In vitro* study design and cell viability analysis from three repeated experiments. Experimental design schematic for *in vitro* treatment with *L. intestinalis* and PA under HFD conditions **(A)**. Cell viability measured by CCK-8 assay following exposure to PA, with and without *L. intestinalis* treatment **(B)**. Data expressed as mean ± SEM of three independent experiments and 12 wells per group. Statistical significance was determined using the Kruskal–Wallis test (*p*-values shown above the graphs) followed by the Mann–Whitney *U*-test; PA, Palmitic acid; CM, conditioned media.

HIEC-6 cells were exposed to 400 μM PA to establish a high-fat diet mimicking condition, as schematically illustrated in Figure 9A. Cells were treated with live *L. intestinalis* or CM to evaluate their potential protective effects against PA-induced lipotoxic stress. PA treatment alone significantly reduced cell viability compared to the control group (Figure 9B). Treatment with *L. intestinalis* significantly restored cell viability relative to that in the PA group, whereas treatment with CM did not show a consistent protective effect (Figure 9B). These *in vitro* experiments were repeated for three times.

## 4 Discussion

Our study demonstrated that HFHFD reshaped the jejunal microbiota in an age- and sex-dependent manner. We focused on jejunal contents to better capture diet-responsive microbial changes in the small intestine. At the phylum level, HFHFD was consistently associated with reduced Firmicutes and increased Bacteroidetes and Verrucomicrobia, accompanied by a decreased F/B ratio. Importantly, this diet-driven signature persisted even when sex was excluded from the analysis, supporting the presence of a core HFHFD effect on the jejunal ecosystem. The F/B ratio also showed negative correlations with jejunal inflammation and hepatic steatosis, suggesting that HFHFD-induced taxonomic shifts may align with pathological progression across the gut–liver axis. Functionally, Firmicutes and Bacteroidetes are frequently discussed as diet-responsive phyla linked to host metabolic and inflammatory phenotypes; however, the biological interpretation of the phylum level F/B ratio is context dependent and should be made cautiously (Clarke et al., 2012). Many Firmicutes members include taxa associated with short-chain fatty acid production and mucosal homeostasis, whereas Bacteroidetes comprise diverse carbohydrate-utilizing taxa that can expand under altered nutrient/bile acid landscapes (Yi et al., 2025). Consistent with this framework, shifts in the abundance of specific Firmicutes or Bacteroidetes taxa have been linked to intestinal inflammatory states (Shen et al., 2018), and inflammatory bowel disease has been associated with reduced Firmicutes and an overall loss of gut microbial diversity (Morgan et al., 2012). In contrast, our previous study in 2-year-old rats fed a high-fat diet showed a significant increase in the F/B ratio based on stool DNA, whereas the present study quantified phylum-level shifts in jejunal contents under HFHFD (Lee et al., 2018). This contrast highlights that the direction of F/B changes may differ by sampling site (stool vs. small bowel contents) and diet composition, and fecal readouts may not directly mirror small intestinal community patterns. In this regard, the HFHFD associated reduction in jejunal Firmicutes and increase in Bacteroidetes may reflect a shift toward an inflammation associated community structure, consistent with the observed associations between reduced F/B ratio, jejunal inflammation, and hepatic steatosis.

Unlike some high-fat diet models that report an increased F/B ratio in feces (Lee et al., 2018; de Noronha et al., 2024; Liu et al., 2024), our HFHFD model showed a reduced F/B ratio in the jejunum. As the microbial biomass in the small bowel is significantly lower than that of the large bowel, resulting in lower sequencing yields (Supplementary Figure 1D; Kastl et al., 2020; Jensen et al., 2023; Shealy et al., 2024), this difference in the F/B ratio might reflect the distinct ecological characteristics of the small bowel compared to the fecal microbiota. Notably, a decreased F/B ratio has been reported in inflammatory bowel disease (Tsai et al., 2025) and was associated with intestinal inflammation, suggesting that similar inflammation-associated microbial imbalances may occur in the jejunum under HFHFD conditions. The decreased F/B ratio may be partly explained by the marked reduction in *L. intestinalis*, a Firmicutes member that showed a diet-dependent decline and negative correlations with increasing jejunal inflammation and hepatic steatosis. Given that the F/B ratio is a composite index, additional Firmicutes and Bacteroidetes taxa are likely to contribute to this pattern; however, the opposing shifts of representative taxa in our dataset provide a plausible ecological basis for the observed F/B reduction in the jejunum under HFHFD conditions.

Beyond broad compositional changes, species-level patterns highlight potentially meaningful functions within the small bowel. HFHFD reduced *Lactobacillus* abundance overall, with *L. intestinalis* showing a significant decrease in young males and aged females. Although *L. intestinalis* showed an overall decreasing tendency under HFHFD, these significant group-specific reductions highlighted age-, sex-, and diet-dependent vulnerability. Consistent with this *Lactobacillus*-centered vulnerability, the *L. reuteri* group also showed a significant HFHFD-associated reduction in young rats, suggesting that HFHFD exposure may preferentially erode key small intestinal *Lactobacillus* lineages. These findings raise the possibility that HFHFD diminishes protective small-intestinal *Lactobacillus* (Pongking et al., 2020; Liu et al., 2023). Supporting this possibility at the species level, *L. intestinalis* has been reported to show a decreasing trend under high-fat feeding in rats and to be negatively correlated with body weight gain and increased fat mass (Lecomte et al., 2015), suggesting that this commensal is sensitive to obesogenic dietary conditions. Notably, *L. intestinalis* has also been reported to confer therapeutic benefits in other disease settings, including the suppression of colorectal tumorigenesis and attenuation of pancreatic fibrosis in experimental models (Sui et al., 2025; Sun et al., 2025). In our study*, L. intestinalis* showed an overall depletion under HFHFD. Although group-wise significance was detected in young males and aged females, analyses focusing on the main diet effect (pooling across sex and/or age) consistently supported reduced *L. intestinalis* abundance under HFHFD (Supplementary Figure 4). Functionally, our *in vitro* model further supported a protective role of *L. intestinalis*, as live *L. intestinalis* (but not its conditioned medium) significantly restored HIEC-6 cell viability under PA-induced lipotoxic stress (Figure 9B). These observations support the interpretation that the HFHFD-associated depletion of *L. intestinalis* may reflect the loss of potentially protective commensals relevant to the gut and systemic metabolic homeostasis. In contrast, *B. caccae* and *B. vulgatus* exhibited diet- and age-linked patterns, which may reflect differential susceptibility of the jejunal ecosystem. *B. caccae* increased in the HFHFD group, predominantly in females. Although *B. caccae* is generally regarded as a commensal bacterium, recent studies have reported its increased abundance in MASLD-associated gut microbiota, suggesting its potentially dependent role in metabolic liver disease (Wang et al., 2024). Diet is a primary determinant of gut microbiota, and age and sex can modulate the magnitude and direction of diet-induced dysbiosis through differences in mucosal immunity and host microbe interactions (Kim and Schiebinger, 2022). Consistently, our previous F344 high-fat diet study demonstrated that microbial responses were more pronounced in aged animals and differed by sex (Lee et al., 2018). In this regard, the female-specific changes observed in our jejunal dataset may indicate sex-dependent differences in small intestinal immune responses or mucosal ecological characteristics under HFHFD conditions, rather than a uniform pathogenic effect. Because diet–age–sex interactions have been less frequently evaluated in jejunal contents than in fecal samples, our findings add anatomical specificity to this emerging area.

Next, we evaluated whether *L. intestinalis* exerted a protective effect using an *in vitro* model. PA, a major saturated fatty acid enriched upon HFD exposure (Fatima et al., 2019; Huang et al., 2025), has been widely used to mimic HFD-associated lipotoxic conditions in cell-based studies (Chu et al., 2019; Müller and Sturla, 2019). In our *in vitro* experiments, supplementation with live *L. intestinalis* partially restored epithelial cell viability after PA exposure, suggesting a protective effect against PA-induced cytotoxicity. These findings provide functional support for our *in vivo* observations of the HFHFD-associated depletion of *L. intestinalis* in the jejunum. In contrast, treatment with *L. intestinalis*-derived CM did not produce consistent protective effects, indicating that the beneficial effects of *L. intestinalis* might depend on direct bacterium-host interactions rather than solely on secreted metabolites. PA has been reported to induce epithelial injury primarily through pro-inflammatory signaling (Snodgrass et al., 2013; Qiu et al., 2022) and the disruption of tight junction proteins (Li et al., 2021). Accordingly, our findings raise the hypothesis that live *L. intestinalis* may protect epithelial cells by modulating PA-perturbed inflammatory and/or barrier-related pathways. Consistent with this notion, prior work reported that *L. intestinalis* can reduce chemokine related inflammatory signaling and improve tight junction proteins genes in a pancreatic fibrosis model (Sui et al., 2025). Because our current in vitro assay focused on cell viability, further studies will be required to directly test these mechanisms under lipotoxic conditions. These results support *L. intestinalis* as a functionally relevant commensal, whose loss under HFHFD may contribute to increased small intestinal vulnerability, and highlight the importance of active host microbe crosstalk in mediating its protective effects.

The increase in Verrucomicrobia at the phylum level may reflect the expansion of *Akkermansia*, including *A. muciniphila*, in the HFHFD group. Although *A. muciniphila* is widely recognized as a beneficial commensal species (Everard et al., 2013; Cani et al., 2022), its increase alongside HFHFD-induced pathology in our jejunal dataset may indicate compensatory expansion in response to altered mucosal or metabolic conditions (Saenz et al., 2023). Alternatively, this pattern may represent a location-specific microbial change in HFHFD-driven remodeling of the small bowel (Noh and Lee, 2020; Zhou et al., 2021).

Beyond compositional shifts, our beta-diversity and LEfSe analyses suggested that HFHFD induced microbial changes in the jejunum, allowing the expansion of opportunistic taxa in an age- and sex-dependent manners (Wu et al., 2025). Notably, sex-dependent differences in the microbial community structure were pronounced at a young age but were attenuated with aging, suggesting that aging constrains microbial diversity and resilience in the small bowel. This attenuation may be partly explained by age-associated shifts in mucosal immunity and epithelial barrier physiology, which could influence microbiome stability/resilience and, in turn, dampen niche-specific community differences in the small bowel (Best et al., 2025; Shao et al., 2025). In addition, age-related changes in sex hormone milieu may influence mucosal immune tone and host–microbe interactions, potentially contributing to the attenuated sex-dependent differences observed in aged animals (Kim, 2022). Specifically, in our previous study (Ha et al., 2025), we observed that serum estrogen levels did not exhibit the expected significant decline with age when measured via ELISA. This finding suggests that peripheral adipose tissue may serve as a critical source of extra-gonadal estrogen through steroid aromatization, especially under conditions of increased adiposity (Kim, 2026). Such localized estrogen production could maintain a certain hormonal milieu even after ovarian function declines, thereby potentially blurring the biological sex gap in older animals and contributing to the observed attenuation of sex-specific microbial signatures. This microbial restructuring was accompanied by functional reprogramming, with HFHFD showing lower predicted representation of multiple metabolism related KEGG pathways in the jejunal microbiota, suggesting a potential reduction in inferred metabolic functional capacity under dietary stress. Consistent with our findings, a previous high-fat diet study also reported reductions in predicted metabolic functional modules (e.g., energy metabolism) in a rodent model (Choi et al., 2024).

## 5 Limitations

This study has several limitations. First, our analysis focused on jejunal microbiota rather than fecal samples, which limits direct comparison with most microbiome studies that rely on stool-based profiling. Notably, as paired fecal samples were not generated in the current experiment due to practical and budgetary constraints, we have explicitly noted this as a study limitation and framed the fecal comparisons as complementary rather than a direct within-study validation. However, this approach was intentionally chosen to capture small intestinal microbial alterations that may be more directly exposed to dietary components and relevant to gut–liver axis signaling. Second, the functional profiles were inferred using predictive metagenomic analysis based on 16S rRNA gene sequencing. Although this approach provides insights into potential functional shifts, these predictions require validation using shotgun metagenomic or metabolomic analyses. Third, the cross-sectional nature of the study precludes a direct causal inference between microbial alterations and HFHFD-induced pathological phenotypes. Although *L. intestinalis* consistently showed diet-, age-, and sex-dependent depletion and was negatively associated with jejunal inflammation and hepatic steatosis, its direct mechanistic contribution to disease progression could not be fully determined from the present data. Fourth, the *L. intestinalis* strain (KCTC, 5052) used in the *in vitro* model differs from the strain found in the rat jejunum samples. This strain was identified through available reference sequences and commercially purchased from KCTC, as ATCC [49335] was not available at the time. While strain-specific effects may exist, the in vitro model remains relevant for understanding the general functional characteristics of *L. intestinalis* in the gut environment. Future studies incorporating more detailed cellular analyses, such as transepithelial electrical resistance measurements and gene expression profiling of inflammatory and tight junction-related markers, are necessary to elucidate these mechanistic pathways. Finally, a limitation of this study is the unequal sample size across groups, as the aged male control group was reduced to *n* = 5 due to age-related mortality during the trial. While our statistical analyses, including Spearman’s rank correlation and Mann–Whitney U-test, do not assume equal group sizes, this imbalance may reduce statistical power and increase uncertainty for estimates involving this subgroup. Although the use of a rat model allowed strict control of diet, age, and sex, the extrapolation of these findings to human patients with MASLD should be performed with caution.

## 6 Conclusion

HFHFD induced remodeling of jejunal microbiota in an age- and sex-dependent manner. HFHFD consistently reduced Firmicutes and increased Bacteroidetes and Verrucomicrobia, leading to a decreased Firmicutes/Bacteroidetes ratio that was negatively associated with jejunal inflammation and hepatic steatosis. At the species level, the HFHFD preferentially depleted *L. intestinalis* while promoting the expansion of opportunistic taxa, highlighting a shift in the small intestinal microbiota under dietary stress. These compositional changes were accompanied by attenuation of sex-dependent microbial differences with aging and reduced predicted metabolic functional capacity. Overall, our findings suggest the importance of jejunal microbiota as a dynamic and diet-sensitive ecosystem that might contribute to age- and sex-specific susceptibility to metabolic liver pathology, with complementary *in vitro* evidence supporting the potential protective role of *L. intestinalis* against lipid-induced epithelial stress.

## Data Availability

All datasets generated in this study have been deposited in the NCBI Sequence Read Archive (SRA) under BioProject PRJNA1393281 and will be released upon publication.

## Supplementary material

SUPPLEMENTARY FIGURE 1 Comparison of jejunal microbial composition and relative abundance according to diet, age, and sex. **(A)** Overall taxonomic composition comparing groups based on diet and age, with sexes combined. **(B)** Comparison among CON groups. **(C)** Taxonomic composition comparing CON and HFHFD groups regardless of age or sex. **(D)** Comparison of OTUs and read counts among four groups (6-week male CON, 6-week female CON, and corresponding groups from stool microbiota data reported in our previous study). **(E)** Relative abundance of the phylum Verrucomicrobia, **(F)** Proteobacteria, and **(G)** Firmicutes/Bacteroidetes (F/B) ratio comparing CON and HFHFD groups irrespective of age or sex. Statistical significance was determined using the Kruskal–Wallis test (*p*-values shown above the graphs) followed by the Mann–Whitney U-test (**p* < 0.05, ***p* < 0.01, ****p* < 0.001).

SUPPLEMENTARY FIGURE 2 Correlations between histological parameters and the relative abundance of major bacterial genera in the jejunal microbiota of rats. Scatter plots show correlations between histological features and the relative abundance of specific bacterial genera. Correlations of *Lactobacillus* with **(A)** jejunal inflammation and **(B)** hepatic steatosis. Correlations of *Bacteroides* with **(C)** jejunal inflammation and **(D)** hepatic steatosis. Correlations of *Akkermansia* with **(E)** jejunal inflammation and **(F)** hepatic steatosis. Statistical correlations were analyzed using Spearman’s rank correlation, and *r*- and *p*-values are indicated on the graphs (*n* = 6 per group, except for 2-year-old male CON, *n* = 5); 6w, 6 weeks; 2yr, 2 years; CON, control; HFHFD, high-fructose high-fat diet.

SUPPLEMENTARY FIGURE 3 Comparison of alpha- and beta-diversity indices in the jejunal microbiota of rats according to diet, age, and sex. Beta-diversity analysis based on principal coordinate analysis (PCoA) plots illustrating microbial community distribution within **(A)** male and **(B)** female groups, including 6-week CON, 6-week HFHFD, 2-year CON, and 2-year HFHFD rats. Pairwise comparisons of microbial composition among the four groups were performed using the PERMANOVA method, and corresponding *p*- and *q*-values are summarized in the tables below each plot (all sets *p*-value = 0.001, shown above the graphs). PC1 explains 31.802% of the variance, PC2 explains 21.624% in **(A)** male rats, and PC1 explains 36.550% of the variance, PC2 explains 10.877% in **(B)** female rats. **(C–F)** Alpha-diversity indices representing **(C)** operational taxonomic units (OTUs), **(D)** Chao1 richness, **(E)** Shannon diversity, and **(F)** phylogenetic diversity in each group (*n* = 6 per group, except for 2-year-old male CON, *n* = 5); 6w, 6 weeks; 2yr, 2 years; CON, control; HFHFD, high-fructose high-fat diet.

SUPPLEMENTARY FIGURE 4 Relative abundance of *Lactobacillus intestinalis* under HFHFD across pooled groupings. **(A)** Relative abundance of *L. intestinalis* stratified by age (young vs aged) and diet (CON vs HFHFD) with sex pooled. Sample sizes: aged CON, *n* = 11; all other groups, *n* = 12. **(B)** Relative abundance of *L. intestinalis* stratified by sex (male vs female) and diet (CON vs HFHFD) with age pooled. Sample sizes: male CON, *n* = 11; all other groups, *n* = 12. **(C)** Relative abundance of *L. intestinalis* stratified by diet only (CON vs HFHFD) with age and sex pooled. Sample sizes: CON, *n* = 23; HFHFD, *n* = 24. Scatter plots display individual animals; data are presented as mean ± SEM. Statistical significance was assessed using the Mann–Whitney U test, and exact *p* values are shown in the plots.
