# Supplementary figures and images for: Age- and sex-dependent alterations of jejunal microbiota in Fischer 344 rats fed with a high-fructose, high-fat diet: depletion of Lactobacillus intestinalis in small bowel contents
Source: Front Microbiol. 2026 Mar 2;17:1779112. doi: 10.3389/fmicb.2026.1779112 (PMC12989582; doi:10.3389/fmicb.2026.1779112)

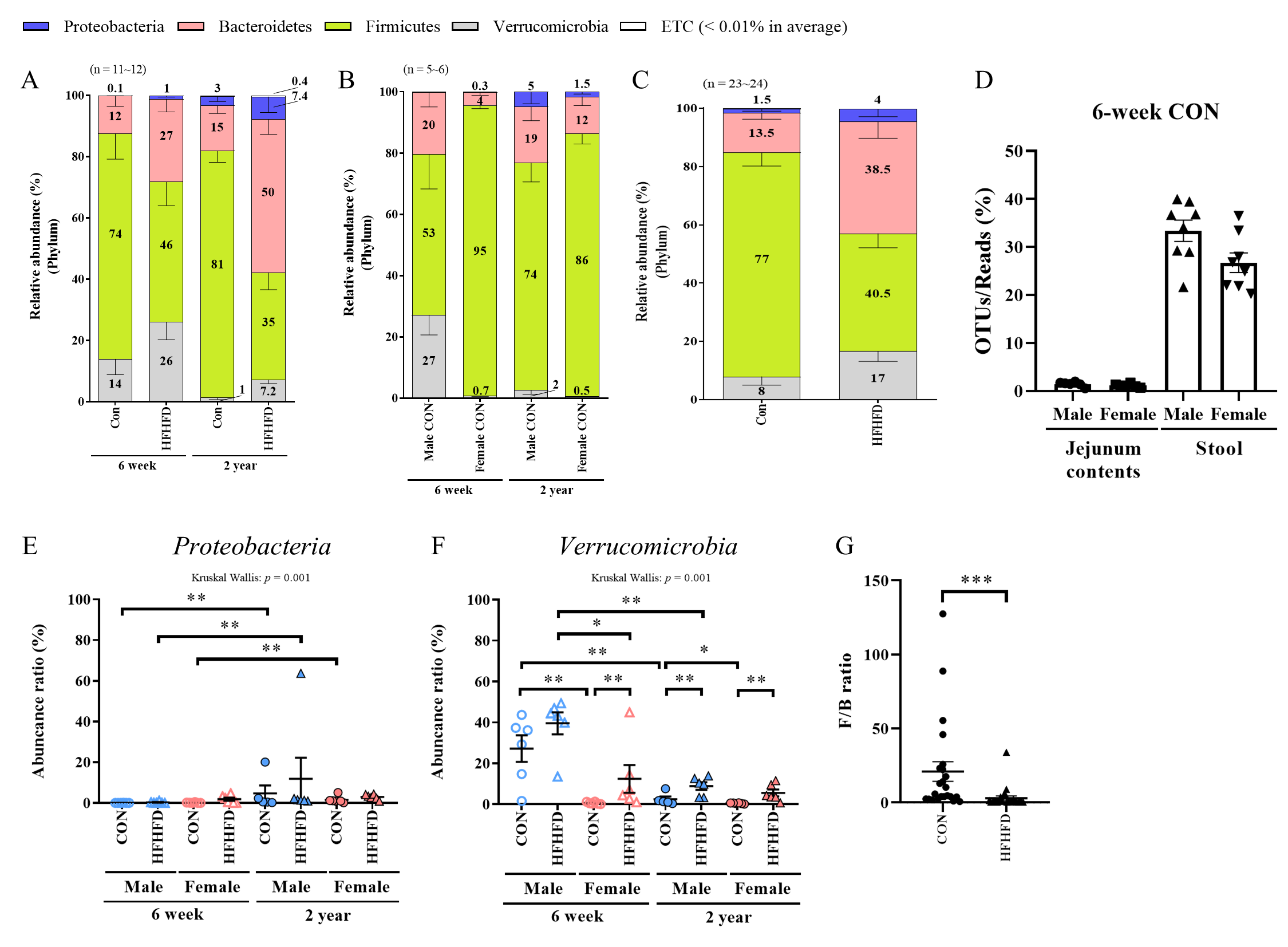

Supplement: SUPPLEMENTARY FIGURE 1 — Comparison of jejunal microbial composition and relative abundance according to diet, age, and sex. (A) Overall taxonomic composition comparing groups based on diet and age, with sexes combined. (B) Comparison among CON groups. (C) Taxonomic composition comparing CON and HFHFD groups regardless of age or sex. (D) Comparison of OTUs and read counts among four groups (6-week male CON, 6-week female CON, and corresponding groups from stool microbiota data reported in our previous study). (E) Relative abundance of the phylum Verrucomicrobia, (F) Proteobacteria, and (G) Firmicutes/Bacteroidetes (F/B) ratio comparing CON and HFHFD groups irrespective of age or sex. Statistical significance was determined using the Kruskal–Wallis test (p-values shown above the graphs) followed by the Mann–Whitney U-test (*p < 0.05, **p < 0.01, ***p < 0.001). [file Image_1.TIF]

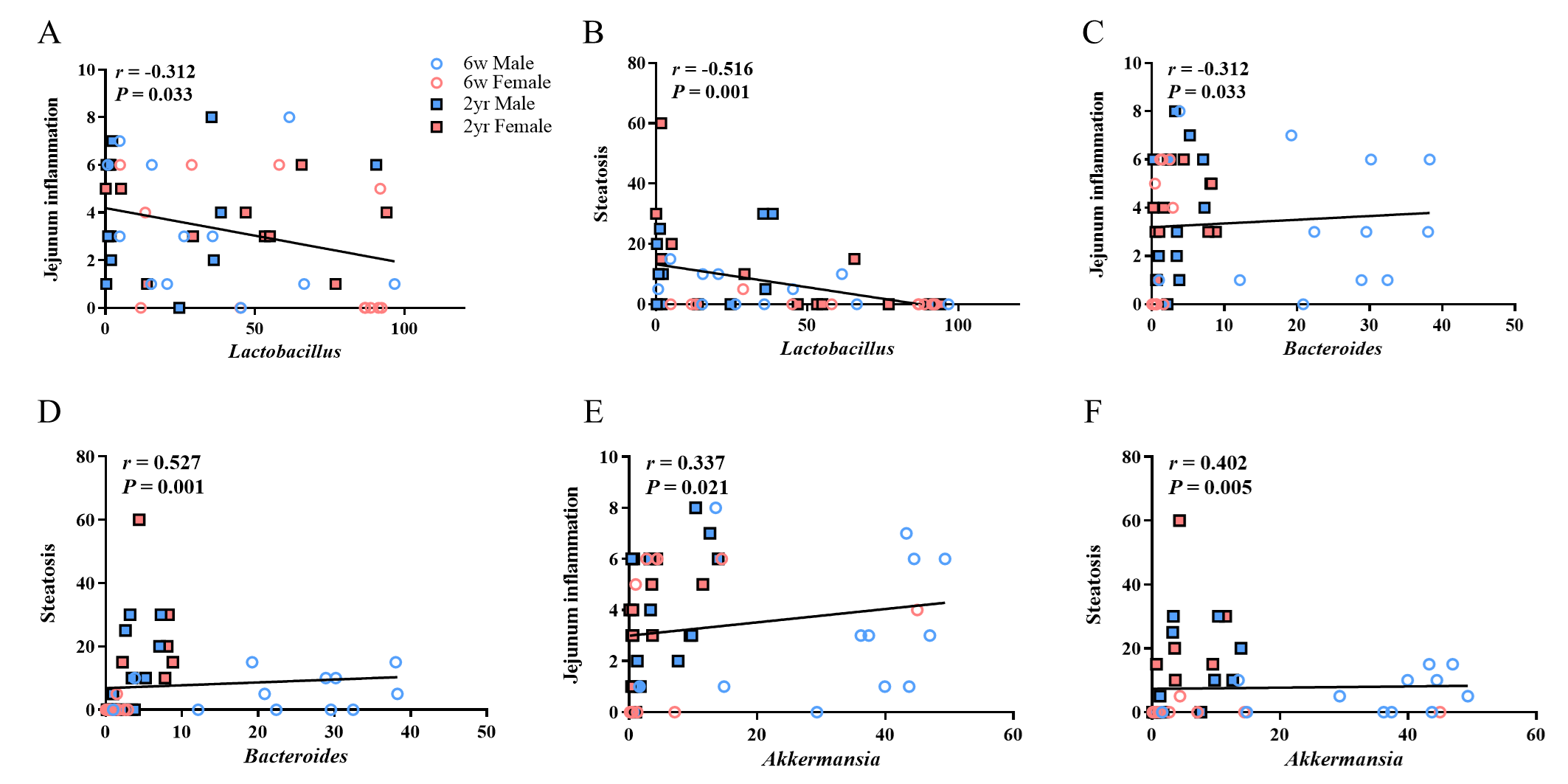

Supplement: SUPPLEMENTARY FIGURE 2 — Correlations between histological parameters and the relative abundance of major bacterial genera in the jejunal microbiota of rats. Scatter plots show correlations between histological features and the relative abundance of specific bacterial genera. Correlations of Lactobacillus with (A) jejunal inflammation and (B) hepatic steatosis. Correlations of Bacteroides with (C) jejunal inflammation and (D) hepatic steatosis. Correlations of Akkermansia with (E) jejunal inflammation and (F) hepatic steatosis. Statistical correlations were analyzed using Spearman’s rank correlation, and r- and p-values are indicated on the graphs (n = 6 per group, except for 2-year-old male CON, n = 5); 6w, 6 weeks; 2yr, 2 years; CON, control; HFHFD, high-fructose high-fat diet. [file Image_2.TIF]

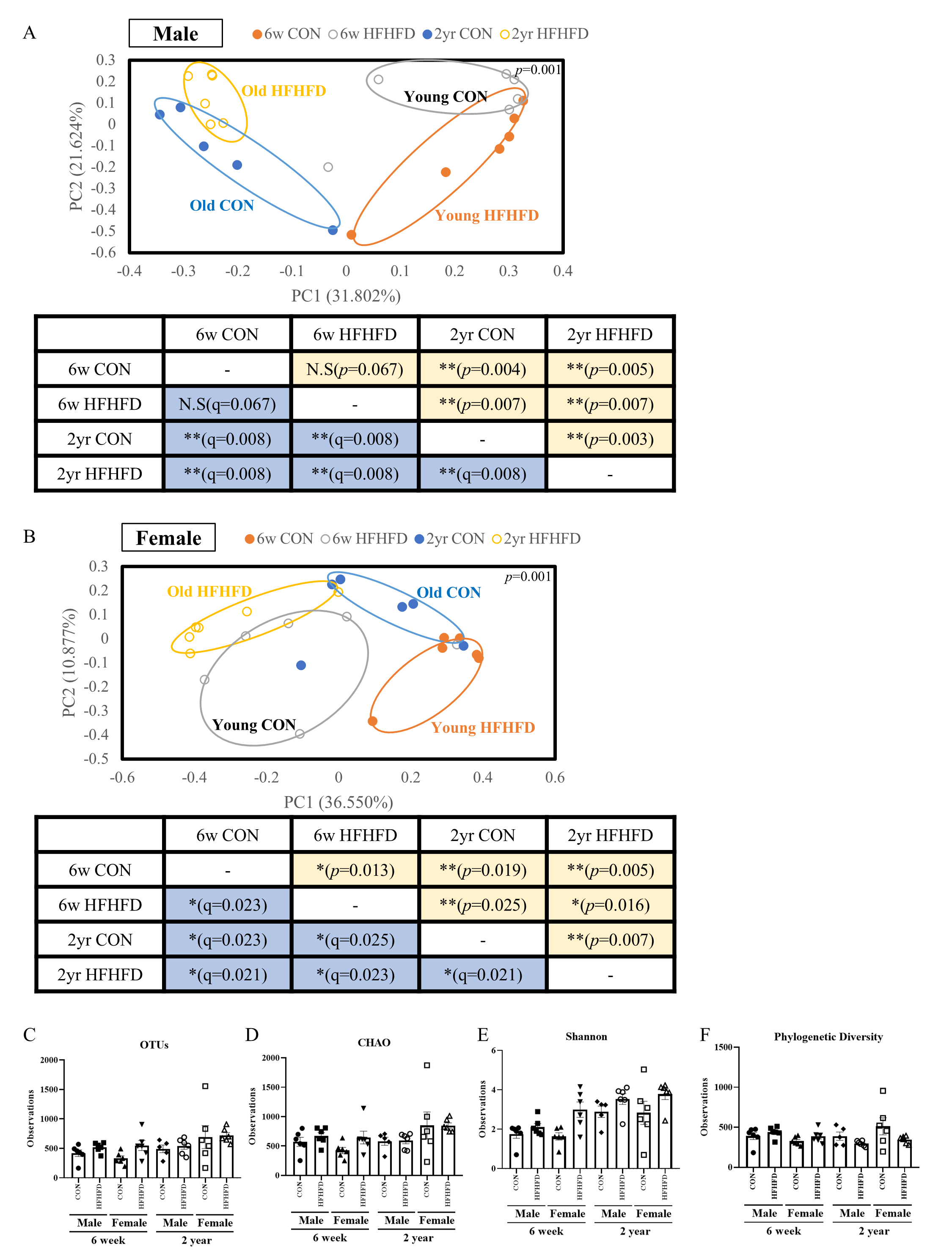

Supplement: SUPPLEMENTARY FIGURE 3 — Comparison of alpha- and beta-diversity indices in the jejunal microbiota of rats according to diet, age, and sex. Beta-diversity analysis based on principal coordinate analysis (PCoA) plots illustrating microbial community distribution within (A) male and (B) female groups, including 6-week CON, 6-week HFHFD, 2-year CON, and 2-year HFHFD rats. Pairwise comparisons of microbial composition among the four groups were performed using the PERMANOVA method, and corresponding p- and q-values are summarized in the tables below each plot (all sets p-value = 0.001, shown above the graphs). PC1 explains 31.802% of the variance, PC2 explains 21.624% in (A) male rats, and PC1 explains 36.550% of the variance, PC2 explains 10.877% in (B) female rats. (C–F) Alpha-diversity indices representing (C) operational taxonomic units (OTUs), (D) Chao1 richness, (E) Shannon diversity, and (F) phylogenetic diversity in each group (n = 6 per group, except for 2-year-old male CON, n = 5); 6w, 6 weeks; 2yr, 2 years; CON, control; HFHFD, high-fructose high-fat diet. [file Image_3.TIF]

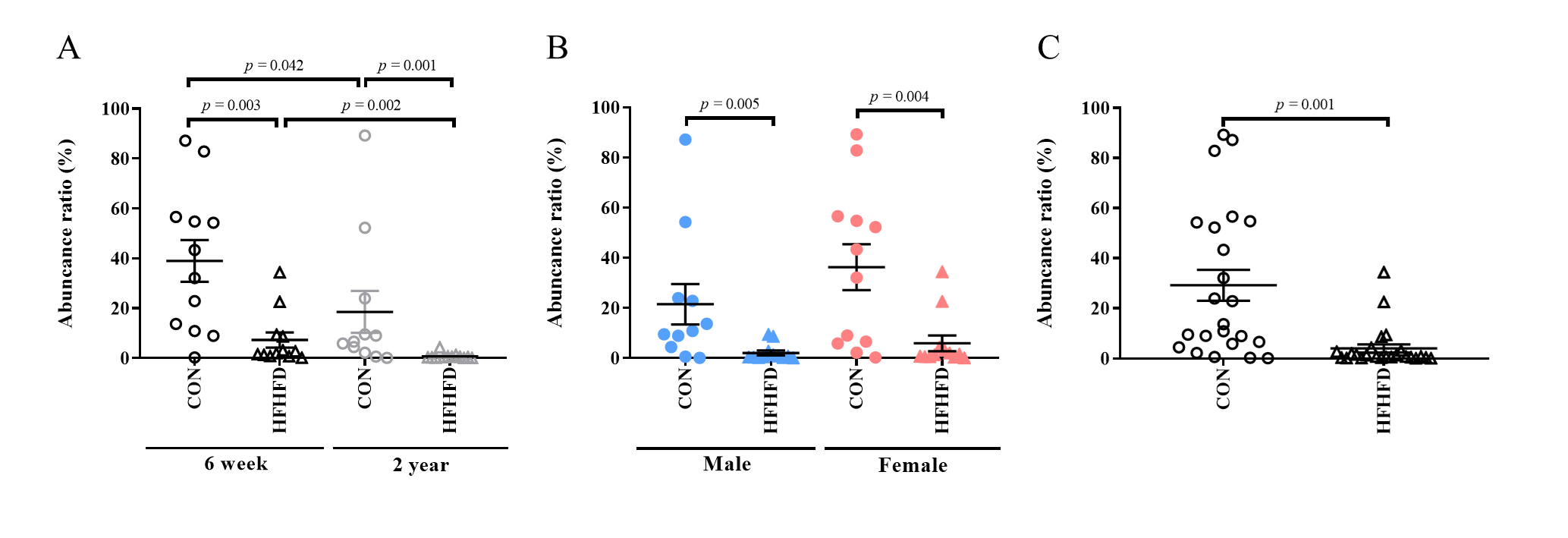

Supplement: SUPPLEMENTARY FIGURE 4 — Relative abundance of Lactobacillus intestinalis under HFHFD across pooled groupings. (A) Relative abundance of L. intestinalis stratified by age (young vs aged) and diet (CON vs HFHFD) with sex pooled. Sample sizes: aged CON, n = 11; all other groups, n = 12. (B) Relative abundance of L. intestinalis stratified by sex (male vs female) and diet (CON vs HFHFD) with age pooled. Sample sizes: male CON, n = 11; all other groups, n = 12. (C) Relative abundance of L. intestinalis stratified by diet only (CON vs HFHFD) with age and sex pooled. Sample sizes: CON, n = 23; HFHFD, n = 24. Scatter plots display individual animals; data are presented as mean ± SEM. Statistical significance was assessed using the Mann–Whitney U test, and exact p values are shown in the plots. [file Image_4.TIF]
